# Supplementary material for: PIMENTO: a primer inference toolkit to facilitate large-scale calling of amplicon sequence variants
Source: Gigascience. 2026 Jul 31;15:giag083. doi: 10.1093/gigascience/giag083 (PMC13508698; doi:10.1093/gigascience/giag083)
Supplement: Supplementary file 2 [file giag083_giga-d-25-00373_revision_1.pdf]

GigaScience

PIMENTO: A primer inference toolkit to facilitate large-scale calling of amplicon sequence variants

--Manuscript Draft--

**Manuscript Number:**	GIGA-D-25-00373R1
**Full Title:**	PIMENTO: A primer inference toolkit to facilitate large-scale calling of amplicon sequence variants
**Article Type:**	Technical Note
**Funding Information:**	European Bioinformatics Institute — Not applicable<br>European Union (101094227, 101112823) — Not applicable
**Abstract:**	The calling of amplicon sequence variants from DNA metabarcoding data is a common method of revealing the taxonomic makeup of environmental samples. A significant hurdle to the large-scale calling of amplicon sequence variants from publicly available nucleotide datasets is the presence of primer sequences in reads, the removal of which is a necessary pre-processing step for this form of analysis. Further, as the details of which primers were used is rarely associated with the sequence records, there is a need for a method that can automatically infer the presence and identity of primers in sequencing data. In this work, we introduce PIMENTO, a Python package which uses a dual-strategy approach for identifying primers that are present in sequencing reads to enable their removal, and therefore facilitate amplicon sequence variant calling at scale.
**Corresponding Author:**	Robert Daniel Finn, PhD<br>EMBL-EBI: European Bioinformatics Institute<br>Hinxton, Cambridge UNITED KINGDOM
**Corresponding Author Secondary Information:**	
**Corresponding Author's Institution:**	EMBL-EBI: European Bioinformatics Institute
**Corresponding Author's Secondary Institution:**	
**First Author:**	Christian Atallah
**First Author Secondary Information:**	
**Order of Authors:**	Christian Atallah<br>Lorna Richardson<br>Martin Beracochea<br>Robert D. Finn
**Order of Authors Secondary Information:**	
**Response to Reviewers:**	Reviewer #1:<br><br>Major comments<br><br>Comment:<br>"""<br>Does PIMENTO perform well on large FASTQ files? Such as those in 1GB sizes after gzip compression. It would be pleased to see some benchmarking data.<br>"""<br>Response:<br>"""<br>A supplementary data with runtime benchmarking results has been added, showing that all PIMENTO functions perform well on larger data.<br>"""

Powered by Editorial Manager® and ProduXion Manager® from Aries Systems Corporation

Comment:
“””
Can PIMENTO be used on Nanopore sequencing data?
“””
Response:
“””
Long-read amplicon sequencing, while growing, is not commonly used, and therefore PIMENTO was not tested on Oxford Nanopore data. Also, typically long-read amplicons go across the whole gene, so primers are not as important to remove. Nonetheless, PIMENTO is marker-gene agnostic, so as long as a primer sequence is consistently at the start of reads, PIMENTO should work.
“””

Comment:
“””
In Paragraph 4, Line 7 of [3.3 Primer cutoff prediction inferences and primer validation strategies]: "in run SRR22098918, a different marine eDNA primer pair was found, which is often compared to the MiFish
universal set[21]."
The primer of SRR22098918 was Teleo (http://dx.doi.org/10.1111/mec.13428). It should be noted that the amplicon length of Teleo is extremely short, only 62bp (excluding primers themselves). Nevertheless, 7 additional bases were predicted (6 in F and 1 in R), which means a removal of 11% (7/62) diversity information after primer trimming. The authors should evaluate whether this would be a major negative impact or not.
“””
Response:
“””
Further discussion on this edge case was written in Section 3.5, at the end of the first paragraph of page 16.
“””

Minor comments

Comment:
“””
Pages and lines are not numbered, making point-to-point comments difficult.
“””
Response:
“””
Page numbers have been added.
“””

Comment:
“””
Paragraph 1, Line 7-8 of [1. Introduction]: "For example, the mitochondrial cytochrome C oxidase subunit I (COI) gene is commonly used to resolve eDNA from macro-organisms like fish and insects"
Fish eDNA researches seldom use COI. Instead, rRNA or Cytb are used more frequently. I suggest to change this example.
“””
Response:
“””
The COI example was kept for the insects, and fish was added to the rRNA example instead, on page 1.
“””

Comment:
“””

Powered by Editorial Manager® and ProduXion Manager® from Aries Systems Corporation

Table 1: Reference databases commonly used in metabarcoding analysis, separated by marker gene.
MIDORI includes all mitochondrial markers rather than COI only. The same is for MitoFish database which could also be added into the table.
""""

Response:
""""

Further detail about MIDORI was added on Table 1 on page 2. MitoFish was also added to Table 1 as a new example database.
""""

Comment:
""""

Paragraph 3, Line 3-6 of [1. Introduction]: Apart from common read QC steps like quality filtering and adapter trimming, the most widely used tools for generating ASVs have an additional strict requirement: the removal of synthetic nucleotides, particularly those coming from amplification primers, including DADA2[9] and deblur[10].
There is duplication here: "adapter trimming" also belongs to "the removal of synthetic nucleotides"
""""

Response:
""""

The duplication was removed on page 2.
""""

Reviewer #2:

Comment:
""""

The title displays the letters PIMENTO in capital letters, which doesn't seem appropriate. It's suggested that the origin of the abbreviation be explained in the abstract or body text.
""""

Response:
""""

The explanation for what PIMENTO stands for was removed from the title, and instead added to the abstract.
""""

Comment:
""""

In "2.1 Standard primer search strategy" part, there is a sentence "This library was curated from a survey of the literature, sequencing platform websites, and other resources." Details should be provided, including how many papers were investigated, the sequencing platform, and what other resources are being referred to.
""""

Response:
""""

The standard primer library was created ad hoc without systematically recording the original source of every primer. However, this comment is noted, and we agree to commit to recording the source of primers added to the library going forward.
""""

Comment:
""""

Figures 2, 3, 4, and 5 can be combined into one figure to make the content more concise, as they all use the Marine Survey Dataset for analysis and present results from different aspects. This also makes it easier for readers to understand.
""""

Powered by Editorial Manager® and ProduXion Manager® from Aries Systems Corporation

	Response:<br>""""<br>This feedback was taken into consideration, however during the writing of this manuscript these figures were originally combined into one, but the authors thought it was harder to interpret, and so the figures were separated. We have kept them separated after these revisions.<br>""""<br><br><br>Comment:<br>""""<br>The box plot in Figure 3 can be further optimized for a more aesthetically pleasing result.<br>""""<br>Response:<br>""""<br>We appreciate the feedback, however aesthetic aside, the authors think the interpretability of the figure is sufficient and therefore does not need changing.<br>""""<br><br><br>Comment:<br>""""<br>The results in the article show that, compared with other types of samples, the differentiation effect of gut microbes is the worst when distinguishing between gut microbes, soil microbes, and marine microbes. The authors explain that this is because there are highly conserved regions at the beginning of the reads. They suggest further analysis or discussion on whether there is a way to identify these highly conserved regions to make the gut microbe samples more accurate.<br>""""<br>Response:<br>""""<br>An extra sentence was added to section 3.3 on page 11 pointing to discussion in section 3.6 about the validation of predicted primers.<br>""""<br><br><br>Comment:<br>""""<br>In "3.5 Primer valiation of automatically-inferred primers" part, suggest authors give an exact example for the validation of the primer inference results.<br>""""<br>Response:<br>""""<br>A new section, 3.4, was created with clear examples of the validation of predicted primers. Section 3.5 has now become 3.6 in the process.<br>""""<br><br>--
**Additional Information:**	
**Question**	**Response**
Are you submitting this manuscript to a special series or article collection?	No
**Experimental design and statistics**<br><br><br>Full details of the experimental design and statistical methods used should be given in the Methods section, as detailed in our	Yes

Minimum Standards Reporting Checklist. Information essential to interpreting the data presented should be made available in the figure legends.<br><br>Have you included all the information requested in your manuscript?	
**Resources**<br><br>A description of all resources used, including antibodies, cell lines, animals and software tools, with enough information to allow them to be uniquely identified, should be included in the Methods section. Authors are strongly encouraged to cite Research Resource Identifiers (RRIDs) for antibodies, model organisms and tools, where possible.<br><br>Have you included the information requested as detailed in our Minimum Standards Reporting Checklist?	Yes
**Availability of data and materials**<br><br>All datasets and code on which the conclusions of the paper rely must be either included in your submission or deposited in publicly available repositories (where available and ethically appropriate), referencing such data using a unique identifier in the references and in the "Availability of Data and Materials" section of your manuscript.<br><br>Have you have met the above requirement as detailed in our Minimum Standards Reporting Checklist?	Yes
GigaScience has policies and guidelines in place for the use of generative AI-writing tools such as ChatGPT. If you have used such writing tools to assist with writing the manuscript this must be declared and cited in the text. Authors should not list AI-writing tools and other	No

AI-assisted technologies as an author or co-author and should acknowledge that they are fully responsible for text generated or refined by AI-writing tools.<p><br><br>A summary of use (particularly in the introduction or among methods) needs to be included at the end of the paper, and the outputs should also be included as a supplementary file hosted in GigaDB or other open repositories. Please read our guidelines for more information. <p><br><br>By submitting to GigaScience, you are aware of the journal's AI-writing tools policy, and if you have declared use of such tools below, you have acknowledged this where appropriate in your manuscript and have made a summary of use and outputs available. <p><br><b>AI-assisted writing tools have been used in the preparation of this manuscript?	

Manuscript
Click here to access/download;Manuscript;PIMENTO_revisions.pdf

PIMENTO: A primer inference toolkit to facilitate large-scale calling of amplicon sequence variants

Christian Atallah[1], Lorna Richardson[1], Martin Beracochea[1], Robert D. Finn[1,*]

[1] European Molecular Biology Laboratory, European Bioinformatics Institute (EMBL-EBI), Wellcome Genome Campus, Hinxton, Cambridge, UK

[*]To whom correspondence should be addressed. Tel: +44 1223 492679; Email: rdf@ebi.ac.uk

Abstract

The identification of amplicon sequence variants from DNA metabarcoding data is a common method for revealing the taxonomic makeup of environmental samples, and for allowing comparative studies between similar datasets. A significant hurdle to the large-scale calling of amplicon sequence variants from publicly available nucleotide datasets is the heterogeneous presence of primer sequences in reads, the removal of which is a necessary pre-processing step for this form of analysis. Furthermore, as the details of the experimental primers are rarely captured in the metadata associated with the sequence records, there is a need for a method that can automatically infer the presence and identity of primers in sequencing data. In this work, we introduce the PrIMER infereNce TOolkit (PIMENTO), a Python package which uses a dual-strategy approach for identifying primers that are present in sequencing reads to enable their removal, and therefore facilitate amplicon sequence variant calling at scale.

Keywords: metagenomics;primer;amplicon;taxonomy;sequencing;quality control

1. Introduction

DNA metabarcoding is a molecular technique that targets a diagnostic marker gene and obtains genetic sequences that can be assigned to a taxonomy. This method is typically applied to genetic material present in environmental samples termed environmental DNA (eDNA), which is increasingly being applied to elucidate the organisms present in an ecosystem**[1,2]**. Depending on the taxonomic group of interest in a metabarcoding experiment, there are different marker genes that are typically chosen for amplification and DNA sequencing. For example, the mitochondrial cytochrome C oxidase subunit I (COI) gene is commonly used to resolve eDNA from macro-organisms like insects, while the ribosomal RNA (rRNA) gene is a typical marker used for microbial diversity resolution, including bacteria and archaea, but even some eukaryotes like fish, fungi and plankton. The use of metabarcoding experiments to reveal the taxonomic composition of samples is popular due to cost-effectiveness, standard protocols and ease of use.

One common approach for the analysis of metabarcoding sequence data is the clustering of reads into high sequence identity groups, termed operational taxonomic units (OTUs). These OTUs can then be taxonomically annotated by comparing to marker-gene-specific databases (Table 1), a process termed closed-reference OTU picking. Closed-reference OTUs therefore help describe taxonomic diversity within a sample, and can be compared across samples when used with a consistent reference database. However, the OTU clustering can easily hide diversity at the lowest taxonomic ranks like species, including unknown organisms that might be of interest. Another approach for the analysis of metabarcoding data, which is growing in popularity, is the calling of amplicon sequence variants (ASVs). ASVs have various benefits compared to OTUs, including more precise resolution down to single-nucleotide differences, and more reliable cross-study comparisons**[10]**, though comparisons can strictly only be made between experiments amplifying the same marker, utilizing a common informatics pipeline.

Table 1: Reference databases commonly used in metabarcoding analysis, separated by marker gene.

**Reference database**	**Marker genes**
SILVA**[3]**	rRNA - SSU and LSU
PR2**[4]**	rRNA - SSU (Protist focused)
Greengenes**[5]**	rRNA - SSU (16S only)
UNITE**[6]**	rRNA - ITS1 and ITS2
MIDORI**[7]**	Mitochondrial markers (COI, 12S)
MitoFish**[8]**	Mitochondrial markers (COI, 12S)
BOLD**[9]**	COI

Like many bioinformatics methods that handle sequencing reads, ASV calling necessitates a degree of quality control (QC) on the reads**[11]**. Apart from common read QC steps like quality filtering, the most widely used tools for generating ASVs have an additional strict requirement: the removal of synthetic nucleotides, particularly those coming from amplification primers, including DADA2**[12]** and deblur**[13]**. Synthetic nucleotides, especially ambiguous bases of primers, are likely to result in high numbers of chimeric ASVs being called, which are made up of two or more abundant "parent" ASVs. As chimeric ASVs are typically filtered out in downstream analyses, it is possible to lose the majority of the data to this filtering step if primer sequences are included in the initial ASV calling, making precise primer trimming necessary for a robust ASV analysis.

Performing primer removal on an individual study basis is usually trivial: the chosen primer sequences are normally known to the scientist conducting the experiment, which are passed to tools like Cutadapt**[14]** for primer removal. However, in larger-scale meta-analysis, where multiple studies may be considered in a single analysis, or where publicly-available data from heterogeneous sources is being analysed, the knowledge of the primer sequences used can become more variable: individual studies might use multiple different primer pairs, primers may or may not have been removed from sequence reads**[15]**. Also, within sequence records in databases like the European Nucleotide Archive (ENA)**[16]**, the experimental metadata attached to a sequence record often does not contain information about used primers**[15]**. Such variation in data availability is problematic for analysis services such as that provided by MGnify**[17]**, where automation of analysis pipelines is necessary. In this case applying the ASV analysis to vast collections of diverse data that are deposited in sequence archives such as ENA, from large studies (like Tara Oceans**[18]** and the Earth Microbiome Project**[19]**) to smaller but more specific studies, can prove more complicated.

Consequently, there is a need for a method to perform automated inference of primer sequences for the purpose of large-scale ASV calling. To this end, we introduce a tool called PIMENTO that implements a dual-strategy method for addressing this important need: it first searches a set of amplicon reads for a predefined list of standard primers that are commonly used in metabarcoding experiments, and if a match is not found a *de novo* prediction for the presence of primers not in the list is performed.

2. Method

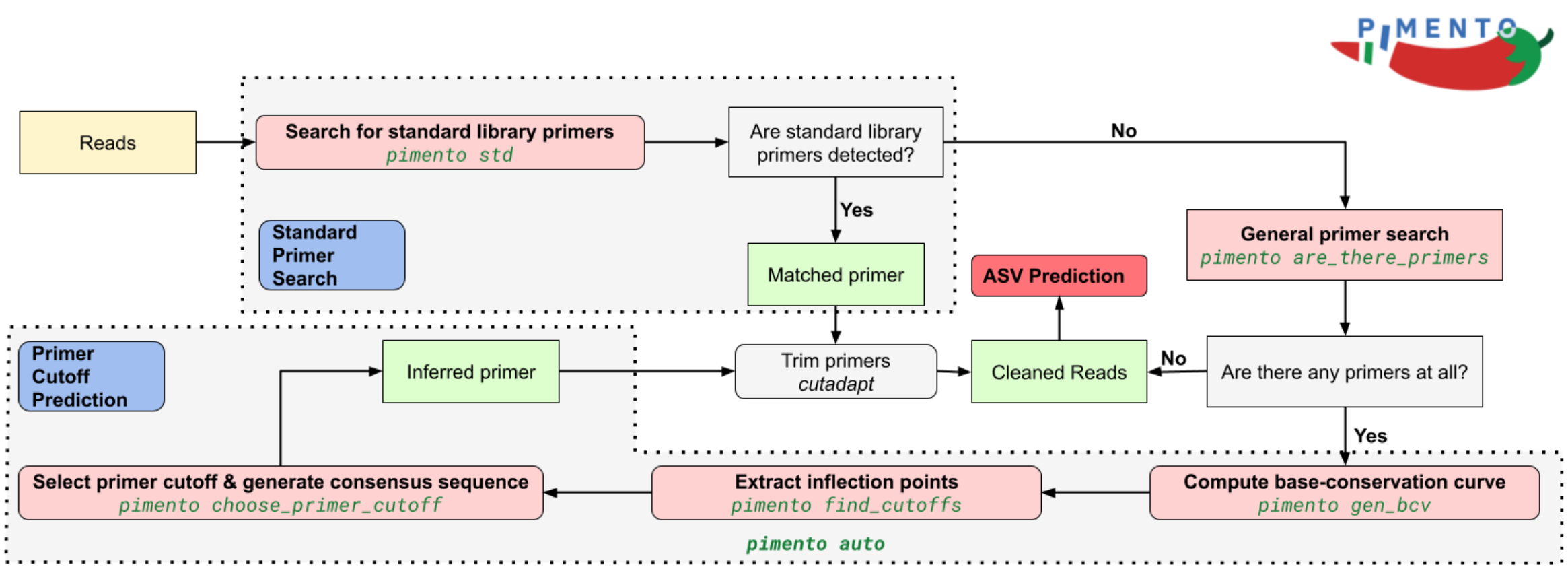

Figure 1: Schematic workflow of the PIMENTO tool. PIMENTO employs a dual primer-search strategy, with the standard library primer search method used first. If primers are found, they can be trimmed from the input reads using a tool like Cutadapt**[14]**. If no primers are matched, the general primer search detects if potential primers are present, then the primer cutoff prediction method is used to

determine a consensus sequence by inferring a cutoff point for the primer to be trimmed.

PIMENTO is a Python command-line tool for performing primer inference on sequencing read files. The source code is available on GitHub**[20]**(https://github.com/EBI-Metagenomics/PIMENTO), and is packaged and released on both PyPi and bioconda for ease of distribution. PIMENTO's dual primer-search strategy (Figure 1) is as follows:

- Standard primer search: fuzzy regular expression search queries to a library of curated standard primer sequences.
- Primer cutoff prediction: identification of the primer cutoff point from analysis of patterns of base conservation at the beginning (and end, for single-end libraries) of reads.

Performance benchmarking for PIMENTO's different functions shows high scalability in its implementation (S1, S2, S3). Below, we explain these two strategies in detail.

2.1 Standard primer search strategy

This first strategy utilises a manually-curated library of standard primers for metabarcoding of the rRNA gene. Typically, specific regions of the rRNA gene are targeted for amplification, including 16S rRNA for both bacteria and archaea, and 18S rRNA and the internal transcribed spacer (ITS1 and ITS2) for eukarya. This library was curated from a survey of the literature, sequencing platform websites, and other resources. The library currently contains 110 primers (names and sequences), of which 55 are forward strand direction (5′ to 3′) and 55 are reverse strand direction (3′ to 5′).

The strategy takes every primer sequence as a query, and performs a search against the first 50 bases of each read, the region where untrimmed primers are expected to reside. The search is fuzzy, allowing for at most two mismatches, to model for sequencing errors, and uses regular expressions, to model for ambiguous bases in some primers. The proportion of reads in which a primer is found is recorded, and any primers with a read proportion lower than a default threshold of 0.60 are disregarded. Once all primers have been searched against the reads, the primer pair with the highest proportion is identified. The default parameters for this step are optimised for the identification of a single pair of primers, but the user can modify this threshold to find multiple sets. However, changing this threshold does not assert the primer pairing, and as the threshold is lowered, the users should take more care in verifying the results.

Corresponding **PIMENTO** command: `pimento std <args>`

2.2 Primer cutoff prediction strategy

The second strategy is based on the analysis of base-conservation patterns at the start of reads to make a prediction on the “cutoff point” of the primer, i.e. where the primer sequence ends and the biological sequence begins. Specifically, it is expected that bases in the “primer space” will have high levels of conservation if the primer has not been removed, as metabarcoding primers are chosen for sequence conservation by design. Complementarily, bases in the immediate “post-primer space” are typically less conserved, as the natural sequence diversity of the organisms in the sample starts revealing itself. However, identifying the cutoff point between the “primer space” and “post-primer space” is complex, as the bases immediately after a primer can still lie in the conserved regions of amplicons like the rRNA gene. Another difficulty is caused by ambiguous bases near the end of a primer sequence, as the patterns of conservation can look similar to a true cutoff point. However, application of the method has demonstrated that the heuristic does select cutoff points that are effective in trimming reads of existing primer sequences.

This approach has one optional but recommended step, and three required steps, all of which use a common concept of “base-conservation”, which we define here. First, a window starting at the i-th base of the sequence and of size n bases is defined, the value of which will vary depending on the step of the method. Second, at each index j of this window, the proportion p of the most commonly-occurring base between A/C/T/G is computed. Finally, the mean base proportion of all the indices of the window is calculated. This mean value will represent the base-conservation BC starting at base i of the window of size n (Equation 1).

$$BC_{i,n} = \frac{\sum_{j=i}^{i+n} p_j}{n}$$

Equation 1: The base-conservation BC of the window starting at base i and of size n is defined as the mean proportion p of the most commonly-occurring base at each index j.

Optional preliminary step

Before running the primer cutoff prediction method, it is optional but recommended to use one of PIMENTO’s utilities to flag as true or false whether there are primers present in the reads. Using base-conservation patterns, this utility splits the first 100 bases of reads into 10 different base-conservation windows of size 10 bases. By comparing the base-conservation of the first two base-pair windows, to the other eight windows, the tool will declare a primer as existing if the bases in those initial two windows are more conserved, based on the sample-specific “average” conservation across all windows.

<u>Corresponding **PIMENTO** command:</u> `pimento are_there_primers <args>`

Step 1
The first step of the primer cutoff prediction method slides a base-conservation window of size 5 across the first 25 indices. The sequence of base-conservation values computed by sliding this window represents a “base-conservation vector” (BCV) for the first 25 bases of the reads.
Corresponding **PIMENTO** command: pimento generate_bcv <args>

Step 2
In the second step, the negative inflection points of this BCV are computed. Every identified negative inflection point, i.e. a point at which the base conservation drops, is considered a potential primer cutoff point that warrants further investigation in the next step.
Corresponding **PIMENTO** command: pimento find_cutoffs <args>

Step 3
In the third and final step, the “best” cutoff point is chosen out of the original set of inflection points. This decision is made based on the difference between base-conservation windows before and after a point - the inflection point that has the largest difference is greedily chosen as the primer cutoff point. As a final output of this process, a consensus sequence is generated based on the previously selected cutoff point. This consensus sequence can then be used downstream to remove the primer from the reads with a tool like Cutadapt.
Corresponding **PIMENTO** command: pimento choose_primer_cutoffs <args>

For ease of use, it is possible to run the three steps of the primer cutoff inference strategy in just one PIMENTO command: pimento auto <args>

3. Results and discussion

To assess the primer inference capabilities of PIMENTO, 855 paired-end, marine microbiome, amplicon sequencing runs were randomly selected and retrieved from the ENA, comprising what we will call the Marine Survey Dataset (MSD) hereafter. The MSD was built by first querying the ENA Browser API with the following query:

```
tax_eq(408172) AND library_strategy="AMPLICON" AND
library_source="METAGENOMIC" AND library_layout="PAIRED"
```

From the result of this query, 855 different studies were randomly chosen, and a random paired-end sequencing run was selected from each study. Thus, the MSD is composed of the 855 pairs of FASTQ files of these selected runs. The different steps

of both the PIMENTO *standard primer search* and *primer cutoff prediction* strategies were then run on the MSD, generating primer inference results for all runs.

As popular choices of primers in metabarcoding experiments depend on the biome being studied, two additional, but smaller biome-specific datasets were built and tested the same way to assess PIMENTO's consistency across different sampling environments. Specifically, a soil survey dataset (SSD) containing 79 random runs was built from ENA, swapping the "tax_eq" value for "410658". Finally, a human gut survey dataset (HGSD) containing 78 runs was built from ENA, swapping the "tax_eq" value for "408170".

3.1 Primer inference outcomes

The results of the *standard primer search* strategy and the optional preliminary step of the *primer cutoff prediction* strategy for all runs of the MSD are summarised in Figure 2. For both forward and reverse FASTQ files, flags were assigned depending on the outcome of each strategy.

- "S" - if a standard primer was found in a direction
- "s" - if no standard primer was found in a direction
- "G" - if a primer was predicted by the cutoff prediction in a direction
- "g" - if no primer was predicted by the cutoff prediction in a direction

For example, if the presence of primers was found to be likely in both directions by step 1 of the *primer cutoff prediction* strategy, and standard primers were found in both directions, the flag "SSGG" was assigned. If only standard primers were found, but the pipeline could not detect the presence of a primer the flag would be "SSgg".

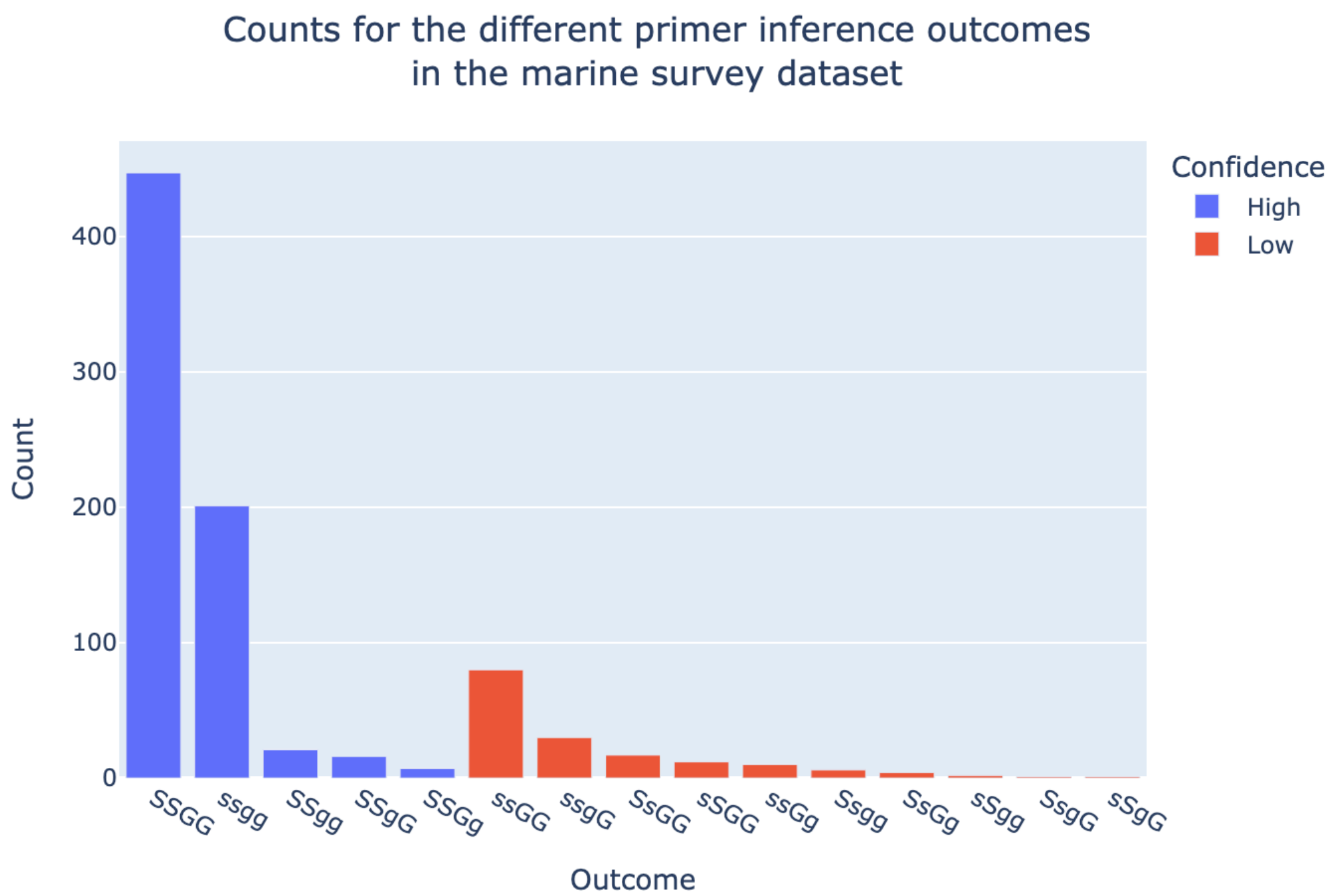

Figure 2: Bar plot showing the distribution of primer inference outcomes for the 855 runs of the Marine Survey Dataset. The outcomes are classified into high (blue) and low (red) confidence depending on the likely accuracy of the outcome category, with 80.9% of the outcomes considered high confidence.

We assessed the different combinations of flags as high and low confidence of being accurate about the presence of primers based on the following interpretations. As the *standard primer search* strategy is more reliable, outcomes where standard primers were found on both strands were considered high confidence. Cases where neither strategies found primers (i.e. ssgg) were also considered high confidence outcomes as both methods indicated a negative presence of primers. All remaining outcomes are considered to be low confidence, as they mostly include cases with negative standard primer presence in combinations that are harder to interpret.

As can be seen in Figure 2, of the 855 runs, 692 were assessed to be high confidence outcomes (80.9%). This number includes 447 runs which were unanimously found to have primers by both methods (i.e. SSGG), while 201 runs (23%) have no primers (i.e. ssgg), indicating that the primers have been removed. Such a low proportion of the runs being confidently primer-free illustrates the problem and shows the current necessity for a tool like PIMENTO.

Low confidence outcomes make up 18.9% of the runs (162 out of 855), with almost half of those (80 out of 162) being from runs where the presence of primers was deemed likely in both directions, but with no matches to standard primers (i.e. ssGG). There are multiple interpretations possible for these cases: the first one - and probably the most likely - is that primers do indeed exist but are not in the standard primer library. For this scenario, the cutoff prediction method will predict an inferred primer to be trimmed, which the method does with high accuracy (see examples in section 3.3). Another possibility is that some runs will be false positives i.e. they have been flagged for primer inference even though there are no primers, which is not implausible due to the use of the heuristic method. Strategies for assessing potential false positives through primer validation are discussed in section 3.5.

3.2 Standard primer inferences

The results in this section pertain to runs with outcomes that were positive for standard primers in both directions (i.e every SS outcome), which total 491 runs. In Figure 3, the distributions of primer presence proportions, in both forward and reverse directions, can be seen. These proportions show that some runs had matching primers as low as 0.61, which still require trimming despite being lower value outliers. Figure 3 also shows the fraction of reads the matched primers were found in, with the first quartiles computing to 0.98 and 0.97, for the forward and reverse directions, respectively. These high values show that for the majority of runs,

matched standard primers were present in the vast majority of reads, which, after trimming, would lead to cleaner reads for ASV calling purposes.

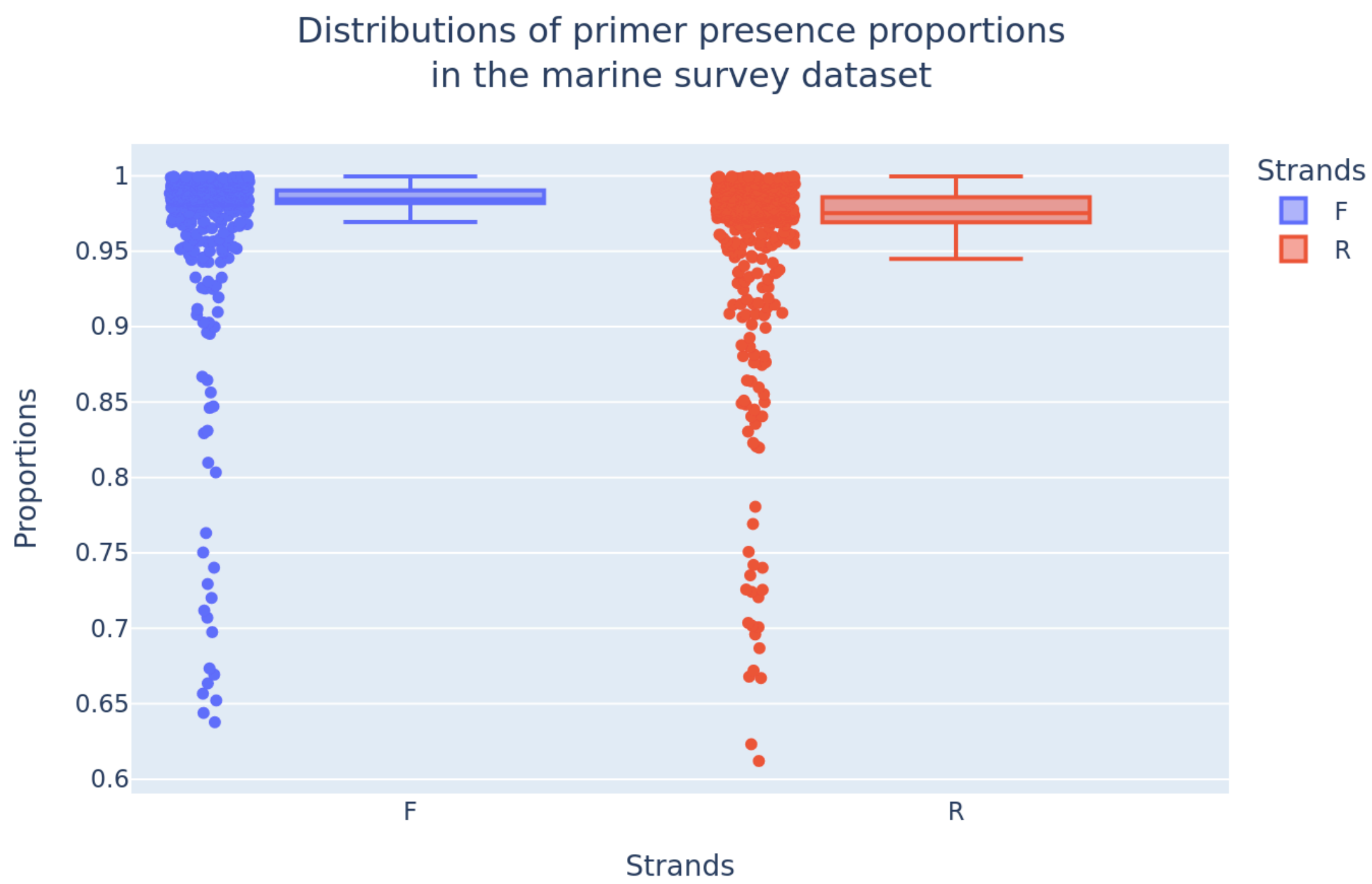

Figure 3: Distribution of the primer presence proportions in the Marine Survey Dataset for matched standard primers for both 5′ to 3′ (F) and 3′ to 5′ (R) directions. The vast majority of the runs had primer proportions in the reads as high as 0.97, with a minority of outliers reaching a proportion as low as 0.61.

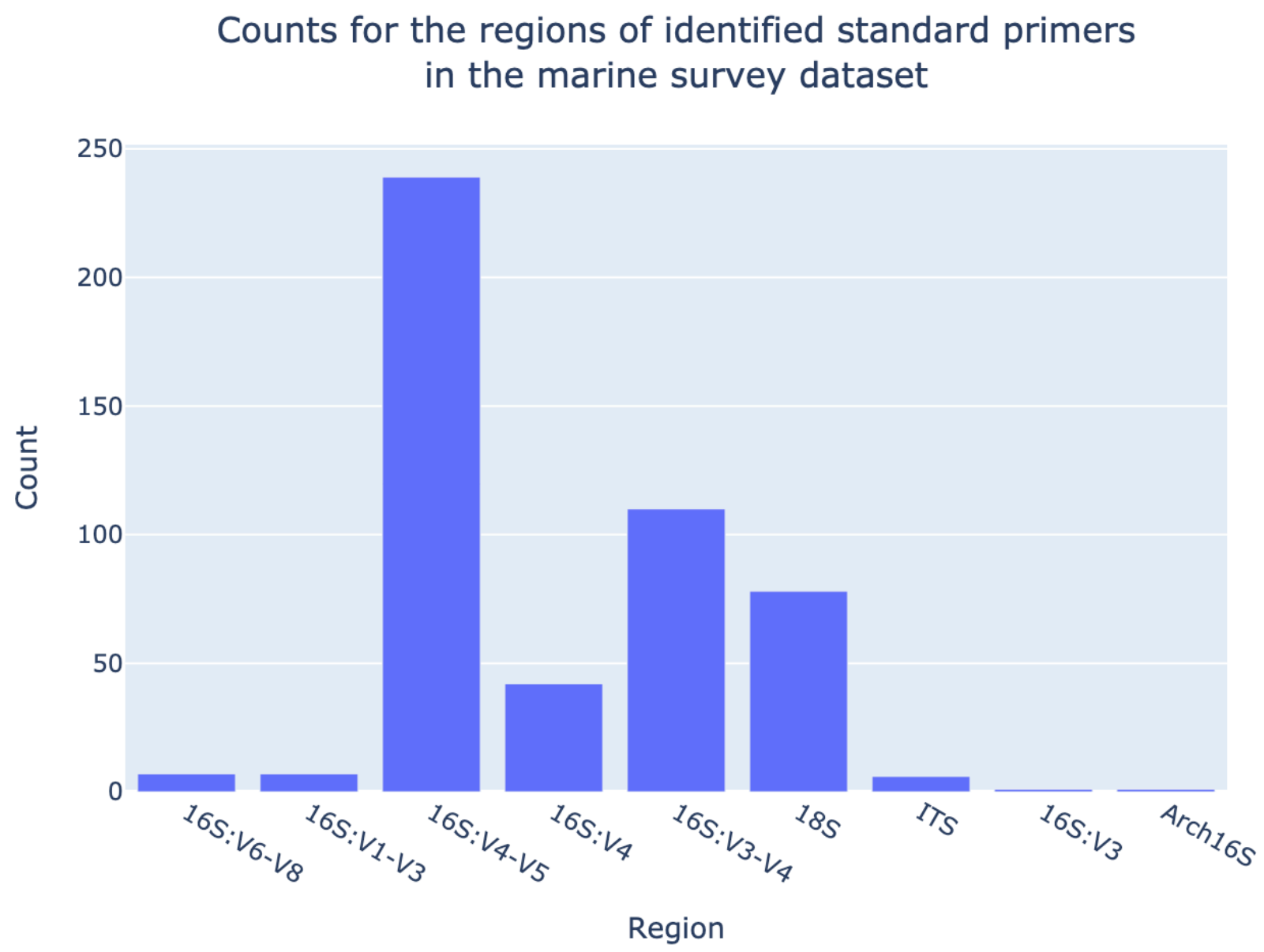

Figure 4: Distribution of identified amplified regions in the Marine Survey Dataset. (A) The most common amplified regions were 16S, but matches to other regions like 18S and ITS were also present.

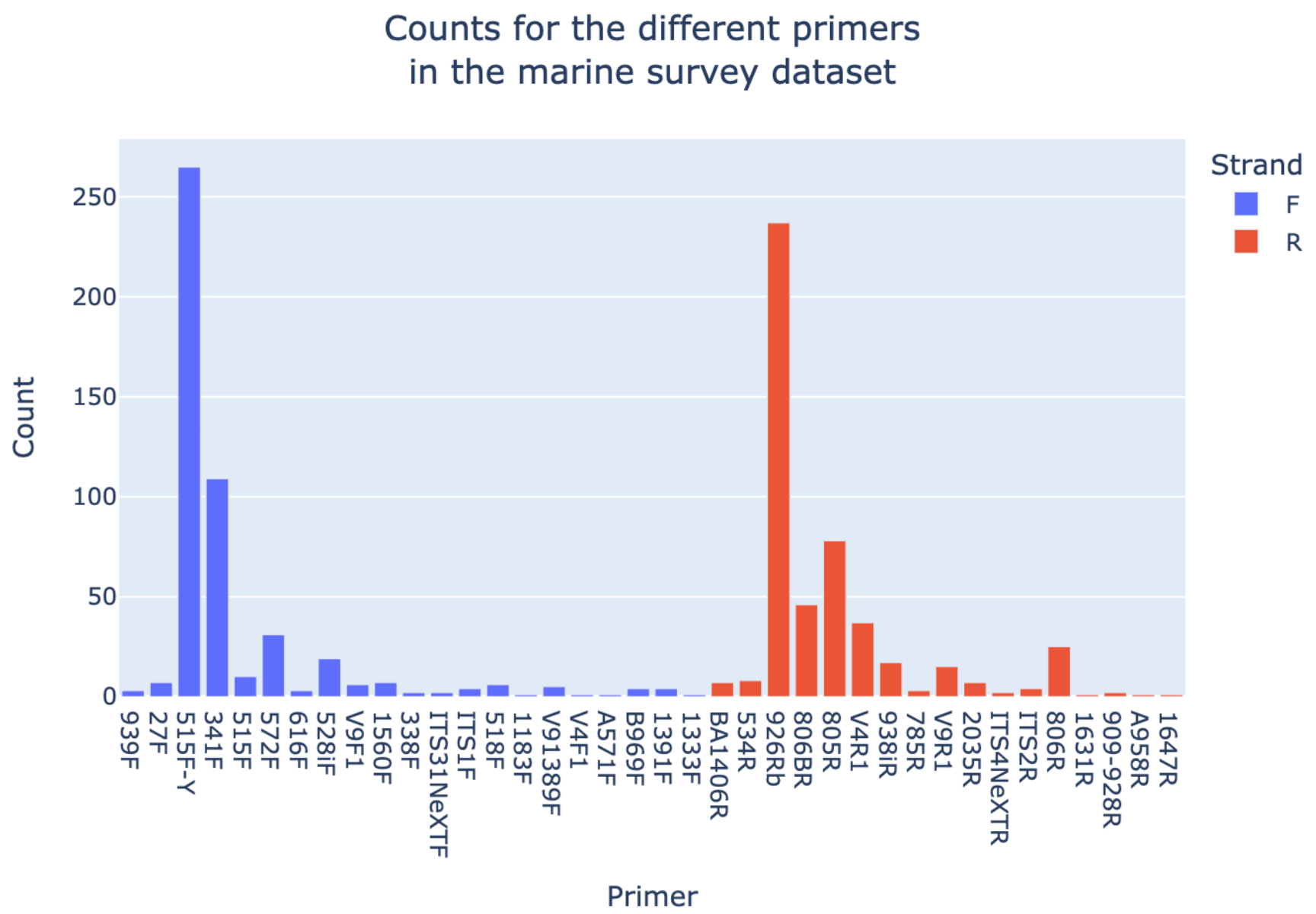

Figure 5: Distribution of identified standard primers in the Marine Survey Dataset for both 5′ to 3′ (F) and 3′ to 5′ (R) directions. As the most common amplified region was 16S (Figure 4), the most common standard primers were also for 16S.

Figure 4 plots the counts for different amplicon regions that were identified by proxy of their standard primer identity. The most common regions are 16S hypervariable regions, which is expected as the most common form of amplicon sequencing. However, the plot also shows the breadth of regions covered by the standard primer library, with occurrences of 18S, ITS, and even Archaea-specific 16S. Figure 5 shows the actual matched primers rather than the regions they amplify. The most common primers tend to be for 16S amplification, but there is a wide range of primers being represented in the MSD.

3.3 Consistency across biomes

The SSD and HGSD datasets were built and analysed by PIMENTO to assess the consistency of performance across different biomes. In Figures 6 and 7, the distribution of outcomes of the *standard primer search* strategy and the optional preliminary step of the *primer cutoff prediction* strategy for the SSD and HGSD can be seen, respectively, using the same interpretation of high and low confidence outcomes as used in section 3.1.

Of the 79 soil runs, 64 were assessed to be high confidence outcomes (81.0%) and 15 were low confidence outcomes (18.9%). These results are very similar as those of the marine dataset, showing consistency across these two biomes. Meanwhile, 53

of the 78 human gut runs were high confidence outcomes (67.9%) and 25 were low confidence outcomes (32%). While these results are still mostly made up of high confidence outcomes, the low confidence outcomes are approximately twice as common in the human gut dataset compared to both the marine and soil datasets. A deeper investigation of these low confidence cases confirmed they are runs where the primers were already removed, but with highly conserved bases at the beginning of reads, leading to false positive primer predictions. This highlights the need to validate the primer predictions where possible. Some strategies for validating primers are discussed in section 3.6.

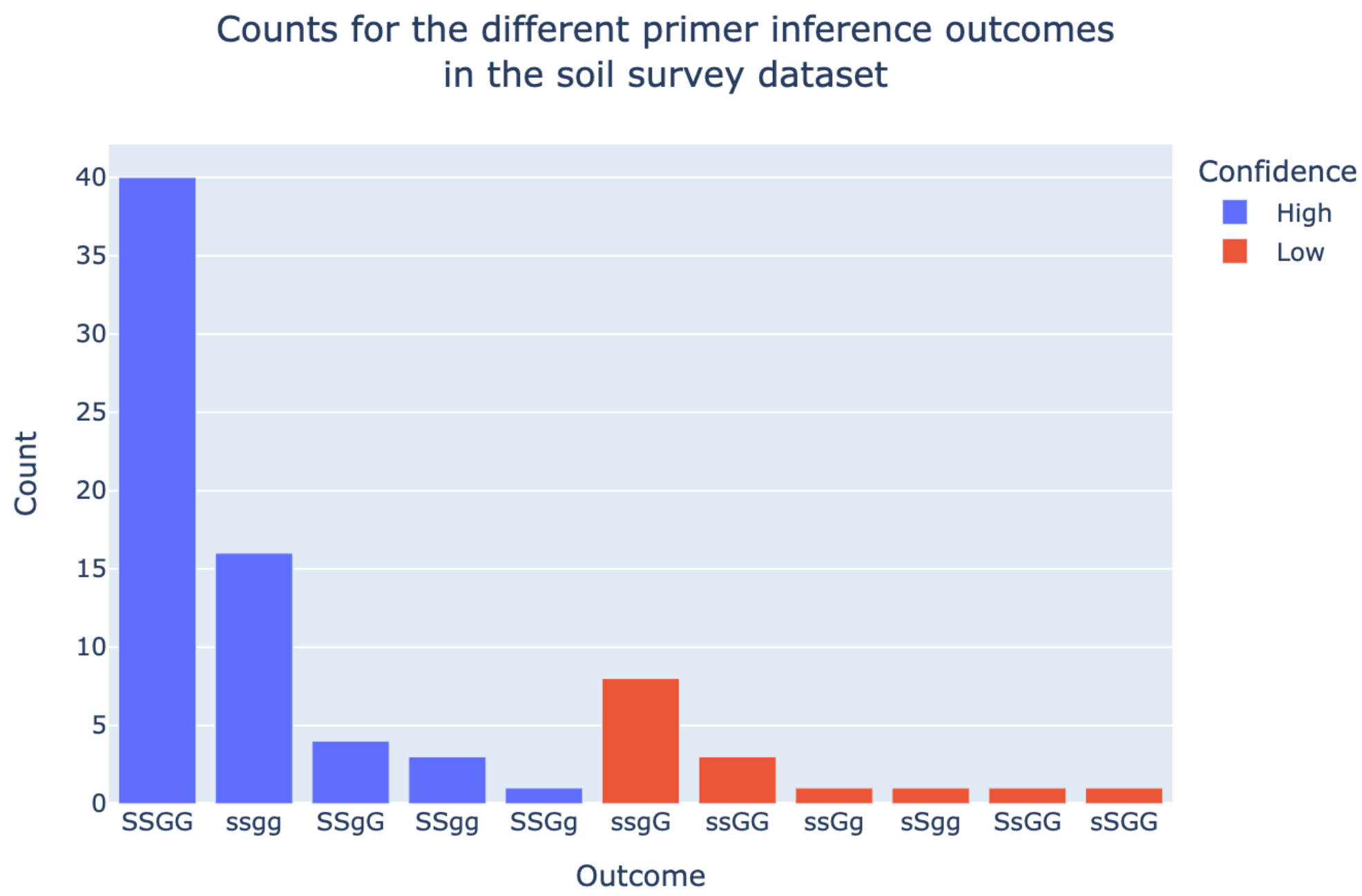

Figure 6: Bar plot showing the distribution of primer inference outcomes for the 79 runs of the Soil Survey Dataset. The outcomes are classified into high, and low confidence as before, with 81.0% of the outcomes considered high confidence.

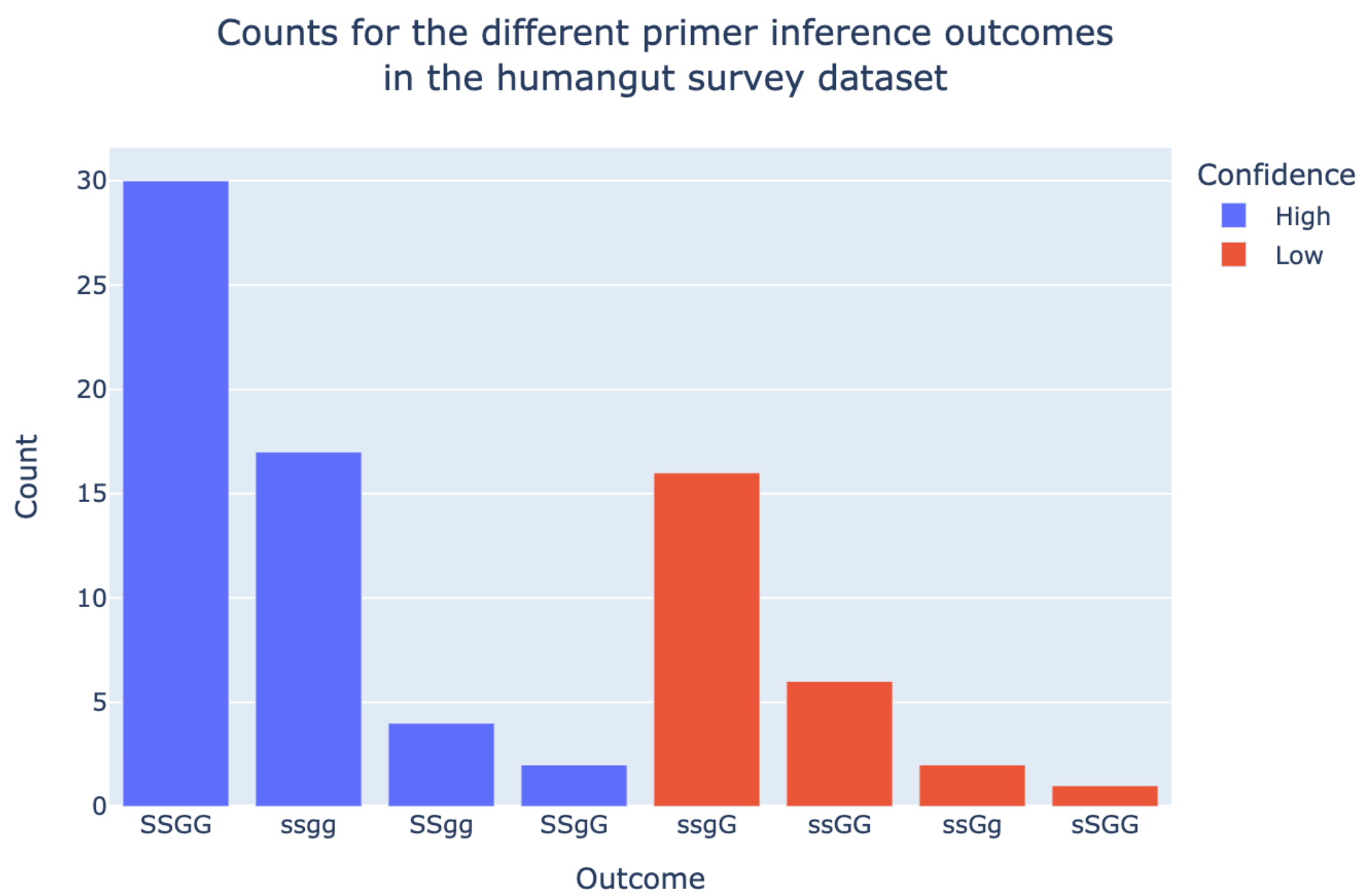

Figure 7: Bar plot showing the distribution of primer inference outcomes for the 78 runs of the Human Gut Survey Dataset. The outcomes are classified into high and low confidence as before, with 67.9% of the outcomes considered high confidence.

3.4 Primer cutoff prediction inferences

The *primer cutoff prediction* inference method was developed as a secondary strategy for cases where primers are not present in the standard primer library bundled with PIMENTO. While it is less reliable than the *standard primer* method due to its heuristic nature, it is applicable to any type of metabarcoding study, and to any type of sequenced amplicon, from rRNA to COI. When a primer is inferred in this way, the end-product is a consensus sequence from the beginning of reads to some chosen optimal cutoff point, and will therefore naturally fit the data it was generated from.

To establish the method's accuracy in predicting real primers, 84 runs from the SSD and HGSD in which standard primers were identified in both orientations were collected. The *primer cutoff prediction* method was used on these 84 runs, generating a total of 168 automatically inferred primers. These inferred primers were then aligned pairwise to their respective standard primers using the Needleman-Wunsch**[21]** global alignment tool.

If the *primer cutoff prediction* method is reliable, we would expect the inferred primers to align well to their standard primer counterparts. Figure 8 shows the distribution of two pairwise alignment metrics: standard primer-based alignment percent identity, which is calculated by dividing the number of matches by the length of the standard primer, and full alignment percent identity, which is calculated by dividing the number of matches by the length of the alignment, including gaps. For the former, the median percent identity is 82.8% for forward primers and 85.0% for reverse primers, while for the latter the percent identity is lower at 69.5% for forward primers and 70.8% for reverse primers. The high percent identity when based on the standard primer lengths shows a high level of precision in the regeneration of real primer sequences by the *primer cutoff prediction* method. However, the full alignment percent identity distribution indicates the presence of gaps, and therefore the presence of extra and/or missing bases in the predicted primers.

Looking closely at three specific examples in Table 2, we can make some assumptions about why the alignment-wide percent identities tend to be lower. For one of the primer pairs of SRR12095443, the alignment percent identity is very high at 95.2%, with only one mismatch. However, the bases coloured in blue reveals that this mismatch is due to the predicted primer having the ambiguous base W, which stands for either A or T, which while less precise, is not a true mismatch.

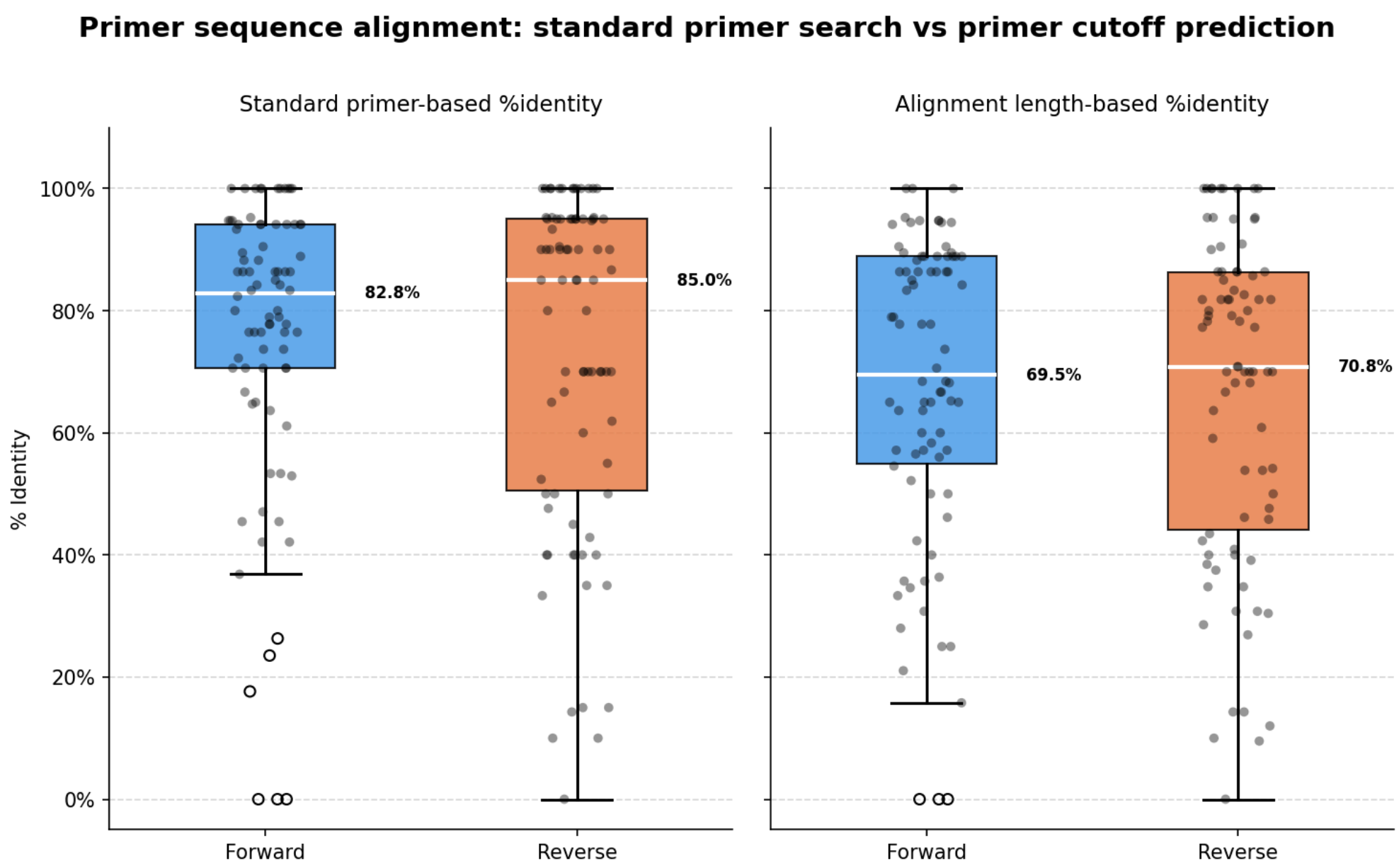

Figure 8: Distribution of two alignment percent identity metrics between sequences identified by the *standard primer search* strategy and the *primer cutoff prediction* method by global pairwise sequence alignment. The standard primer-based percent identity metric is calculated by dividing the number of matches by the length of the standard primer, and the full alignment-based percent identity is calculated by dividing the number of matches by the length of the alignment, including gaps. The former shows that the *primer cutoff prediction* method regenerates standard primers at a high level of identity, while the latter shows that the alignments do have gaps, and therefore extra and missing bases.

For one of the SRR12580015 primer pairs, the alignment percent identity is slightly lower than the median at 65.2%. There are two potential reasons for the lower quality of this alignment. The first is that the predicted primer is two bases shorter than the standard primer, creating a gap at the end. This is due to the heuristic nature of the *primer cutoff prediction* method resulting in a cutoff point two bases shorter than expected. The second, larger issue, is the presence of five bases at the beginning of the predicted primer that do not exist in the standard primer, four of which are the ambiguous base H (A, C, or T), creating a gap. Given that the inferred primer is a consensus sequence generated from the input reads, these bases do actually exist in the reads, and are not spurious. One possibility is that these extra nucleotides are other synthetic bases like primer-tags, adapter sequences, flow-cell binding sequences or multiplex barcodes, which would need to be trimmed for ASV generation, so their presence in the inferred primer is not problematic. All other bases are identical except for one true mismatch from C to G, which could be explained by the use of a slightly different primer than the standard one.

Table 2: Comparison of sequences identified by the *standard primer search* strategy and the *primer cutoff prediction* method by global pairwise sequence alignment. The alignment percent identity is calculated by dividing the number of matching bases by

the length of the alignment, gaps included. These three examples show a high level of precision in regenerating standard primers by the cutoff prediction method, although gaps are present due to missing or extra bases. The final example shows the importance of quality control before running PIMENTO to remove additional artificial bases that were added for sequencing.

Run	Primer Alignment	Alignment %id
SRR12095443	`GACTACHVGGGTATCTAATCC Standard`<br>`|||||||||||| ||||||||`<br>`GACTACHVGGGTWTCTAATCC Predicted`	95.2%
SRR12580015	`     CATCGATGAAGAACGCAG Standard`<br>`     |||||||||||||| ||`<br>`HHHHGCATCGATGAAGAAGGC    Predicted`	65.2%
SRR21642156	**All reads:**<br>`GACTACHVGGGTATCTAATCC Standard`<br>`      ||  |`<br>`DVVHHHHVVRGKDW        Predicted`<br><br>**Reads starting with primer:**<br>`GACTACHVGGGTATCTAATCC Standard`<br>`|||||||||||||||||||||`<br>`GACTACHVGGGTATCTAATCCT Predicted`	All reads: 14.2%<br><br>Reads starting with primer: 95.5%

The final example, which is one of the primer pairs of SRR21642156, had a very low alignment percent identity of just 14.2% - there are only three identical matches, and a sizeable seven base-pair gap due to missing bases at the end of the predicted primer. However, the predicted primer is mostly made up of ambiguous bases, all of which are in agreement with the standard primer bases e.g. the first G being replaced with a D, which stands for either A, G, or T. Investigating the start of these reads, the reason is likely due to extra bases of variable lengths at the beginning of most reads. Out of 49,990 reads, only 14,323 start with the standard primer, 14,151 start with TG, 11,257 start with AC, 7,780 start with CTA, and 7 start with just T.

The *standard primer* method handles reads like these, since primers are searched for anywhere at the start of reads. However, this experiment does highlight a limitation of the *primer cutoff prediction* method, in that it generates primers through the consensus of reads once a cutoff point has been selected, which is adversely affected by varying starts to reads. Because the pre-primer length of these reads are variable, the base distributions of the primer are not in register, leading to ambiguous bases at every position. Nonetheless, if these extra bases are trimmed beforehand, which they would be at the QC stage of most bioinformatics pipelines, the *primer cutoff prediction* method would handle cases like these correctly. In fact, running the method on just the reads that start with the primer resulted in the prediction of the primer ‘GACTACHVGGGTATCTAATCCT’, which when aligned to

the standard primer results in an alignment percent identity of 95.5% (Table 2), indicating that QC would allow for correct primer inference.

3.5 Retrieval of automatically inferred primers from literature

Multiple examples of inferred primers from the MSD were retrieved and compared to matches found in literature, which can be seen in Table 3. In run DRR059938 shows a Large Subunit (LSU) primer pair - LSUCop-D2 - which has been used in marine metagenomics analysis using the D2 region of the LSU as a marker**[22]**. Both the forward and reverse primers map to the native sequences with exact identity and coverage, with the inferred reverse primer being slightly longer by two bases.

In run SRR6378319, a standard primer for the 16S V3 region was found that was not originally in the curated library - BV3r**[23]**. In this run, the forward primer was an existing standard primer for the V3 region: 341F. This is an example of an outcome where a standard primer was found in just one direction, but primers were predicted to exist in both directions (i.e. SsGG). In this case a standard primer was simply missing from the library, and has since been added to it. The predicted primer overlaps with 100% identity and coverage, although it does over extend by three bases.

Table 3: Comparison of four primer sequences that were predicted by the *primer cutoff prediction* method and its native counterparts, found in literature. The alignment between the native and predicted primer sequences are coloured in green. All four examples show a high coverage, and exact sequence identity for said coverage, with some gaps.

Run	Primer Alignments	Alignment %id
DRR059938 **[22]**	**F:**<br>`AGACCGATAGCAAACAAGTAC` **Literature**<br>`|||||||||||||||||||||`<br>`AGACCGATAGCAAACAAGTAC` **Predicted**<br>**R:**<br>`GTCCGTGTTTCAAGACGG` **Literature**<br>`||||||||||||||||||`<br>`GTCCGTGTTTCAAGACGGGT` **Predicted**	F: 100%<br>R: 100%
SRR6378319 **[23]**	**R:**<br>`CCGCGGCTGCTGGCAC` **Literature**<br>`||||||||||||||||`<br>`CCGCGGCTGCTGGCACGDA` **Predicted**	R: 84.2%
SRR13172663 **[24]**	**F:**<br>`GTGTCGGTAAAACTCGTGCCAGC` **Literature**<br>`|||||||||||||||||||||||`<br>`GTGTCGGTAAAACTCGTGCCAGC` **Predicted**	F: 100%<br>R: 74.1%

	R:<br>CATAGTGGGGTATCTAATCCCAGTTTG Literature<br>|||||||||||||||||||| CATAGTGGGGTATCTAATCC Predicted	
SRR22098918 **[25]**	F:<br>ACACCGCCCGTCACTCT Literature<br>||||||||||||||||| ACACCGCCCGTCACTCTCCCCAA Predicted<br>R:<br>CTTCCGGTACACTTACCRTG Literature<br>||||||||||||||||| || CTTCCGGTACACTTACCATGT Predicted	F: 73.9%<br>R: 90.5%

In run SRR13172663, a primer pair for marine eDNA studies**[24]** was found in run SRR13172663. This pair is actually a universal set for fish eDNA metabarcoding, with the forward primer matching perfectly. The reverse primer is however missing 7 bases out of 20, which is explained by the cutoff prediction strategy preferring to select shorter primers as part of its algorithm, as to avoid trimming biological bases and losing potential real diversity in the process. Finally, in run SRR22098918, a different marine eDNA primer pair was found, teleo, which is often compared to the MiFish universal set**[25]**. Sequence identity was again exact, with a small number of extended bases for both forward and reverse directions (6 and 1 respectively), and one ambiguous base being (R) replaced by a non-contradicting standard base (A). However, the amplicon targeted by the teleo primers is usually around 65bp in length**[26]**. Due to the short size of this amplicon, the removal of 6 native bases could adversely impact the resulting ASV analysis more strongly. This impact could be mitigated by setting a maximum primer length downstream of the *primer cutoff prediction* method's results.

These results show that the *primer cutoff prediction* strategy for inference is marker-agnostic. While the MSD was created by retrieving sequencing runs that were annotated as originating from microbial metabarcoding experiments, incorrect annotations in public databases resulted in some eDNA studies being selected, for which primers were still able to be inferred. A further aim was to identify sequences that could be considered for addition to the standard library, such as the V3 BV3r primer. It is encouraging to note that on every occasion demonstrated in these results, the primers matched with exact sequence identity to the native primers, with no gaps or mismatches. Nonetheless, the inferred primers are not perfect: some are longer than the native primer, which could result in removal of biological diversity, and some are shorter, which could still impact ASV calling results as the primer is not fully removed. Also, there are likely to be false positives, as some runs will have been flagged for primer inference when unnecessary.

3.6 Primer validation of automatically-inferred primers

As was discussed in sections 3.3 and 3.5, one potential source of error in PIMENTO is the false positive inference of primers existing in reads when it is not the case. To prevent such false positives, validating low confidence automatically-inferred primers is always recommended. This validation can be performed either by referring to existing literature that describes the primer, or by computational means. For example, using a tool like Infernal/cmsearch with the different Rfam covariance rRNA models as input e.g. RF00177 for the bacterial 16S region, one could make sure that the generated primer sequence lies within coordinates that we would expect for a primer, i.e. before or after a hypervariable region, rather than within one.

4. Conclusion

In this work, we have introduced PIMENTO, which is a set of Python scripts that implement a novel dual-strategy method for filling an important gap in large-scale ASV analysis of metabarcoding data. Using either a curated library of standard primers to search against, or base-conservation patterns to automatically predict good cutoff points for inferring a consensus sequence, we believe this toolkit will help enable analysis pipelines that generate ASVs at scale.

As was shown in sections 3.4 and 3.5, the automatically inferred primers generated by the cutoff-based method have exact sequence identity to native primers, but can sometimes be either slightly shorter or longer than the correct sequence. During the course of this work, multiple primers that were not originally in the standard primer library, but were predicted as existing and then generated by the *primer cutoff prediction* method, were subsequently added to the standard primer library, including the BV3r primer that was discussed in section 3.5. The addition of further primers to the library when they are first encountered means that future runs using them will benefit, as they will now be detected by the standard primer strategy directly, leading to more precise primer trimming. There is therefore significant potential in the crowdsourcing of further additions to the standard primer library, e.g. through the creation of new issues on PIMENTO's GitHub repository, naturally improving PIMENTO's detection capabilities over time.

Source code, data, and package availability

The source code for PIMENTO is publicly available on GitHub at this link: https://github.com/EBI-Metagenomics/PIMENTO. The files making up the standard primer library can be found in the repository at this link: https://github.com/EBI-Metagenomics/PIMENTO/tree/main/pimento/standard_primers. Finally, PIMENTO is available on both PyPi and bioconda as the "mi-pimento" package.

List of abbreviations

eDNA: environmental DNA; COI: cytochrome C oxidase subunit I; rRNA: ribosomal RNA; OTUs: operational taxonomic units; ASVs: amplicon sequence variants; QC: quality control; ENA: European Nucleotide Archive; MSD: Marine Survey Dataset; SSD: soil survey dataset; HGSD: human gut survey dataset; LSU: Large Subunit;

Funding

Funded by the European Union under the Horizon Europe Programme with Grant Agreement No. 101112823 (DTO-BioFlow) and 101094227 (Blue-Cloud 2026). Views and opinions expressed are however those of the author(s) only and do not necessarily reflect those of the European Union or the European Climate, Infrastructure and Environment Executive Agency (CINEA). Neither the European Union nor the granting authority can be held responsible for them. This project is also funded by European Molecular Biology Laboratory core funds.

References

[1] P. Taberlet, E. Coissac, F. Pompanon, C. Brochmann, and E. Willerslev, "Towards next-generation biodiversity assessment using DNA metabarcoding," *Molecular Ecology*, vol. 21, no. 8, pp. 2045–2050, Apr. 2012, doi: 10.1111/j.1365-294x.2012.05470.x.

[2] E. Aßmann, S. Agrawal, L. Orschler, S. Böttcher, S. Lackner, and M. Hölzer, "Impact of reference design on estimating SARS-CoV-2 lineage abundances from wastewater sequencing data," *GigaScience*, vol. 13, 2024, doi: 10.1093/gigascience/giae051.

[3] C. Quast *et al.*, "The SILVA ribosomal RNA gene database project: improved data processing and web-based tools," *Nucleic Acids Research*, vol. 41, no. D1, pp. D590–D596, Nov. 2012, doi: 10.1093/nar/gks1219.

[4] L. Guillou *et al.*, "The Protist Ribosomal Reference database (PR2): a catalog of unicellular eukaryote Small Sub-Unit rRNA sequences with curated taxonomy," *Nucleic Acids Research*, vol. 41, no. D1, pp. D597–D604, Nov. 2012, doi: 10.1093/nar/gks1160.

[5] D. McDonald *et al.*, "An improved Greengenes taxonomy with explicit ranks for ecological and evolutionary analyses of bacteria and archaea," *The ISME Journal*, vol. 6, no. 3, pp. 610–618, Dec. 2011, doi: 10.1038/ismej.2011.139.

[6] R. H. Nilsson *et al.*, "The UNITE database for molecular identification of fungi: handling dark taxa and parallel taxonomic classifications," *Nucleic Acids Research*,

vol. 47, no. D1, pp. D259–D264, Oct. 2018, doi: 10.1093/nar/gky1022.
[7] M. Leray, N. Knowlton, and R. J. Machida, “MIDORI2: A collection of quality controlled, preformatted, and regularly updated reference databases for taxonomic assignment of eukaryotic mitochondrial sequences,” *Environmental DNA*, vol. 4, no. 4, pp. 894–907, Apr. 2022, doi: 10.1002/edn3.303.
[8] W. Iwasaki *et al.*, “MitoFish and MitoAnnotator: A Mitochondrial Genome Database of Fish with an Accurate and Automatic Annotation Pipeline,” *Molecular Biology and Evolution*, vol. 30, no. 11, pp. 2531–2540, Aug. 2013, doi: 10.1093/molbev/mst141.
[9] S. Ratnasingham and P. D. N. Hebert, “bold: The Barcode of Life Data System (http://www.barcodinglife.org),” *Molecular Ecology Notes*, vol. 7, no. 3, pp. 355–364, Jan. 2007, doi: 10.1111/j.1471-8286.2007.01678.x.
[10] B. J. Callahan, P. J. McMurdie, and S. P. Holmes, “Exact sequence variants should replace operational taxonomic units in marker-gene data analysis,” *The ISME Journal*, vol. 11, no. 12, pp. 2639–2643, Jul. 2017, doi: 10.1038/ismej.2017.119.
[11] Y. Gao *et al.*, “Benchmarking short-read metagenomics tools for removing host contamination,” *GigaScience*, vol. 14, 2025, doi: 10.1093/gigascience/giaf004.
[12] B. J. Callahan, P. J. McMurdie, M. J. Rosen, A. W. Han, A. J. A. Johnson, and S. P. Holmes, “DADA2: High-resolution sample inference from Illumina amplicon data,” *Nature Methods*, vol. 13, no. 7, pp. 581–583, May 2016, doi: 10.1038/nmeth.3869.
[13] A. Amir *et al.*, “Deblur Rapidly Resolves Single-Nucleotide Community Sequence Patterns,” *mSystems*, vol. 2, no. 2, Apr. 2017, doi: 10.1128/msystems.00191-16.
[14] M. Martin, “Cutadapt removes adapter sequences from high-throughput sequencing reads,” *EMBnet.journal*, vol. 17, no. 1, p. 10, May 2011, doi: 10.14806/ej.17.1.200.
[15] K. Kuhbandner, “FAIR enough? A perspective on the status of nucleotide sequence data and metadata on public archives,” The Company of Biologists, Nov. 2021. Accessed: Aug. 19, 2025. [Online]. Available: https://doi.org/10.1242/prelights.30958
[16] R. Leinonen *et al.*, “The European Nucleotide Archive,” *Nucleic Acids Research*, vol. 39, no. Database, pp. D28–D31, Oct. 2010, doi: 10.1093/nar/gkq967.
[17] L. Richardson *et al.*, “MGnify: the microbiome sequence data analysis

resource in 2023," *Nucleic Acids Research*, vol. 51, no. D1, pp. D753–D759, Dec. 2022, doi: 10.1093/nar/gkac1080.
[18] C. Bowler, "Tara Oceans Expedition sequences the ocean," *TheScienceBreaker*, vol. 01, no. 04, Nov. 2015, doi: 10.25250/thescbr.brk004.
[19] J. A. Gilbert, J. K. Jansson, and R. Knight, "The Earth Microbiome project: successes and aspirations," *BMC Biology*, vol. 12, no. 1, Aug. 2014, doi: 10.1186/s12915-014-0069-1.
[20] EBI-Metagenomics, "GitHub - EBI-Metagenomics/PIMENTO: A PrIMEr infereNce TOolkit to facilitate large-scale calling of metabarcoding amplicon sequence variants," GitHub. Accessed: Mar. 21, 2025. [Online]. Available: https://github.com/EBI-Metagenomics/PIMENTO
[21] S. B. Needleman and C. D. Wunsch, "A general method applicable to the search for similarities in the amino acid sequence of two proteins," *Journal of Molecular Biology*, vol. 48, no. 3, pp. 443–453, Mar. 1970, doi: 10.1016/0022-2836(70)90057-4.
[22] S. Wu *et al.*, "DIFFERENCES OF COPEPODA COMPOSITION ANALYSIS IN MARICULTURE PONDS THROUGH MORPHOLOGICAL TAXONOMY AND DIFFERENT METAGENETIC APPROACHES," *Applied Ecology and Environmental Research*, vol. 21, no. 3, pp. 2091–2131, 2023, doi: 10.15666/aeer/2103_20912131.
[23] K. Sahm *et al.*, "High abundance of heterotrophic prokaryotes in hydrothermal springs of the Azores as revealed by a network of 16S rRNA gene-based methods," *Extremophiles*, vol. 17, no. 4, pp. 649–662, May 2013, doi: 10.1007/s00792-013-0548-2.
[24] M. Miya *et al.*, "MiFish, a set of universal PCR primers for metabarcoding environmental DNA from fishes: detection of more than 230 subtropical marine species," *Royal Society Open Science*, vol. 2, no. 7, p. 150088, Jul. 2015, doi: 10.1098/rsos.150088.
[25] R. Brys *et al.*, "Monitoring of spatiotemporal occupancy patterns of fish and amphibian species in a lentic aquatic system using environmental DNA," *Molecular Ecology*, vol. 30, no. 13, pp. 3097–3110, Dec. 2020, doi: 10.1111/mec.15742.
[26] J. Yang, L. Zhang, Y. Mu, and X. Zhang, "Small changes make big progress: A more efficient eDNA monitoring method for freshwater fish," *Environmental DNA*, vol. 5, no. 2, pp. 363–374, Jan. 2023, doi: 10.1002/edn3.387.

Click here to access/download
Supplementary Material
PIMENTO_supplementary.pdf
