# Supplementary material for: PIMENTO: a primer inference toolkit to facilitate large-scale calling of amplicon sequence variants
Source: Gigascience. 2026 Jul 31;15:giag083. doi: 10.1093/gigascience/giag083 (PMC13508698; doi:10.1093/gigascience/giag083)
Supplement: Supplementary file 5 [file giag083_reviewer_1_report_original_submission.pdf]

Reviewer Report

Title: PIMENTO: A primer inference toolkit to facilitate large-scale calling of amplicon sequence variants

Version: Original Submission **Date:** 11/4/2025

Reviewer name: Reviewer 1

Reviewer Comments to Author:

********* Overall comments *********PIMENTO is a wonderful tool to help users solve the problem when primers' information are unknown. I think the methods are right, but I sightly doubted on the necessity.For a certain metabarcoding dataset without primers' information, the most straightforward way is to consult the authors, which is the only way to get true results. In the manuscript, the authors have been clarified that PIMENTO could be used in automatically pipelines dealing with larger-scale meta-analysis. However, I am afraid that "automatically" larger-scale meta-analysis is challenging since many other factors need to be considered such as negative and/or positive controls, biological and/or technical replications. Similar with primer sequences, such information usually not appear in the meta-data, and need to refer to the original publications or authors, and I suggest the authors to have some discussion on this point.********* Major comments *********1. Does PIMENTO perform well on large FASTQ files? Such as those in 1GB sizes after gzip compression. It would be pleased to see some benchmarking data.2. Can PIMENTO be used on Nanopore sequencing data?3. In Paragraph 4, Line 7 of [3.3 Primer cutoff prediction inferences and primer validation strategies]: "in run SRR22098918, a different marine eDNA primer pair was found, which is often compared to the MiFishuniversal set[21]."The primer of SRR22098918 was Teleo (http://dx.doi.org/10.1111/mec.13428). It should be noted that the amplicon length of Teleo is extremely short, only 62bp (excluding primers themselves). Nevertheless, 7 additional bases were predicted (6 in F and 1 in R), which means a removal of 11% (7/62) diversity information after primer trimming. The authors should evaluate whether this would be a major negative impact or not.********* Minor comments *********1. Pages and lines are not numbered, making point-to-point comments difficult.2. Paragraph 1, Line 7-8 of [1. Introduction]: "For example, the mitochondrial cytochrome C oxidase subunit I (COI) gene is commonly used to resolve eDNA from macro-organisms like fish and insects"Fish eDNA researches seldom use COI. Instead, rRNA or Cytb are used more frequently. I suggest to change this example.3. Table 1: Reference databases commonly used in metabarcoding analysis, separated by marker gene.MIDORI includes all mitochondrial markers rather than COI only. The same is for MitoFish database which could also be added into the table.4. Paragraph 3, Line 3-6 of [1. Introduction]: Apart from common read QC steps like quality filtering and adapter trimming, the most widely used tools for generating ASVs have an additional strict requirement: the removal of synthetic nucleotides, particularly those coming from amplification primers, including DADA2[9] and deblur[10].There is duplication here: "adapter trimming" also belongs to "the removal of synthetic nucleotides"

Level of Interest

Please indicate how interesting you found the manuscript: Choose an item.

Quality of Written English

Please indicate the quality of language in the manuscript: Choose an item.

Declaration of Competing Interests

Please complete a declaration of competing interests, considering the following questions:

- Have you in the past five years received reimbursements, fees, funding, or salary from an organisation that may in any way gain or lose financially from the publication of this manuscript, either now or in the future?
- Do you hold any stocks or shares in an organisation that may in any way gain or lose financially from the publication of this manuscript, either now or in the future?
- Do you hold or are you currently applying for any patents relating to the content of the manuscript?
- Have you received reimbursements, fees, funding, or salary from an organization that holds or has applied for patents relating to the content of the manuscript?
- Do you have any other financial competing interests?
- Do you have any non-financial competing interests in relation to this paper?

If you can answer no to all of the above, write 'I declare that I have no competing interests' below. If your reply is yes to any, please give details below.

I declare that I have no competing interests

I agree to the open peer review policy of the journal. I understand that my name will be included on my report to the authors and, if the manuscript is accepted for publication, my named report including any attachments I upload will be posted on the website along with the authors' responses. I agree for my report to be made available under an Open Access Creative Commons CC-BY license (http://creativecommons.org/licenses/by/4.0/). I understand that any comments which I do not wish to be included in my named report can be included as confidential comments to the editors, which will not be published.

Choose an item.

To further support our reviewers, we have joined with Publons, where you can gain additional credit to further highlight your hard work (see: https://publons.com/journal/530/gigascience). On publication of this paper, your review will be automatically added to Publons, you can then choose whether or not to claim your Publons credit. I understand this statement.

Yes Choose an item.
