# Supplementary material for: PIMENTO: a primer inference toolkit to facilitate large-scale calling of amplicon sequence variants
Source: Gigascience. 2026 Jul 31;15:giag083. doi: 10.1093/gigascience/giag083 (PMC13508698; doi:10.1093/gigascience/giag083)
Supplement: Supplementary file 6 [file giag083_reviewer_1_report_revision_1.pdf]

Reviewer Report

Title: PIMENTO: A primer inference toolkit to facilitate large-scale calling of amplicon sequence variants

Version: Revision 1 **Date:** 4/30/2026

Reviewer name: Reviewer 1

Reviewer Comments to Author:

The authors did not use a tracking system (or highlight changes) to show the changes they made in the revised manuscript, which makes it hard for reviewers to check whether their comments were addressed. I suggest the authors use a tracking system in the future revision.*********************************Previous Comment 1:PIMENTO is a wonderful tool to help users solve the problem when primers' information are unknown. I think the methods are right, but I sightly doubted on the necessity.For a certain metabarcoding dataset without primers' information, the most straightforward way is to consult the authors, which is the only way to get true results. In the manuscript, the authors have been clarified that PIMENTO could be used in automatically pipelines dealing with larger-scale meta-analysis. However, I am afraid that "automatically" larger-scale meta-analysis is challenging since many other factors need to be considered such as negative and/or positive controls, biological and/or technical replications. Similar with primer sequences, such information usually not appear in the meta-data, and need to refer to the original publications or authors, and I suggest the authors to have some discussion on this point.Authors' Response: NoneNew Comment:Not sure if the authors have missed this comment, but I did not see any revision or discussion on this point.*********************************Previous Comment 2:Can PIMENTO be used on Nanopore sequencing data?Authors' ResponseLong-read amplicon sequencing, while growing, is not commonly used, and therefore PIMENTO was not tested on Oxford Nanopore data. Also, typically long-read amplicons go across the whole gene, so primers are not as important to remove. Nonetheless, PIMENTO is marker-gene agnostic, so as long as a primer sequence is consistently at the start of reads, PIMENTO should work.New Comment:No, Nanopore sequencing is not equal to long-read amplicon sequencing. There are already several researches using Nanopore data for short amplicons (such as the ~170bp MiFish universal set). Followings are some examples:Doorenspleet, K., Jansen, L., Oosterbroek, S., Kamermans, P., Bos, O., Wurz, E., Murk, A. and Nijland, R. (2025), The Long and the Short of It: Nanopore-Based eDNA Metabarcoding of Marine Vertebrates Works; Sensitivity and Species-Level Assignment Depend on Amplicon Lengths. Mol Ecol Resour, 25: e14079. https://doi.org/10.1111/1755-0998.14079 (the short amplicon part)Cahyani, N. K. D., A. W.Anggoro, R.Prasetia, et al. 2026. "eDNA Metabarcoding as a Promising Conservation Tool to Monitor Fish Diversity in Indonesia Marine Protected Areas." Environmental DNA8, no. 1: e70242. https://doi.org/10.1002/edn3.70242.Jansen, L., E.Loos, R. W.Ariyati, et al. 2025. "A Baseline of Fish Species Richness Through eDNA Metabarcoding in an Understudied Tropical Mangrove Coast of Java, Indonesia." Environmental DNA7, no. 6: e70201. https://doi.org/10.1002/edn3.70201.Cartairade, L., J.Poulain, Q.Carradec, S.Mangenot, P.Wincker, and S.Planes. 2026. "Optimizing a Novel eDNA-Based Framework for Reef Fish Biodiversity Monitoring Using

an Autonomous Filtration System and in situ Nanopore Sequencing." Ecology and Evolution16, no. 3: e73254. https://doi.org/10.1002/ece3.73254.I still hope the authors can test PIMENTO on the above Nanopore data.**********************************Others:I have noticed a lot of grammar errors, such as "it is possible to have a run with less reads but more diversity having a higher runtimethan a run with more reads but less diversity, which is what we see in these plots." from Page 3 in the supplementary materials. The phrase "less reads" should be "fewer reads". I suggest the authors to have a native English speaker to proofread all the files.

Level of Interest

Please indicate how interesting you found the manuscript: Choose an item.

Quality of Written English

Please indicate the quality of language in the manuscript: Choose an item.

Declaration of Competing Interests

Please complete a declaration of competing interests, considering the following questions:

- Have you in the past five years received reimbursements, fees, funding, or salary from an organisation that may in any way gain or lose financially from the publication of this manuscript, either now or in the future?
- Do you hold any stocks or shares in an organisation that may in any way gain or lose financially from the publication of this manuscript, either now or in the future?
- Do you hold or are you currently applying for any patents relating to the content of the manuscript?
- Have you received reimbursements, fees, funding, or salary from an organization that holds or has applied for patents relating to the content of the manuscript?
- Do you have any other financial competing interests?
- Do you have any non-financial competing interests in relation to this paper?

If you can answer no to all of the above, write 'I declare that I have no competing interests' below. If your reply is yes to any, please give details below.

I declare that I have no competing interests

I agree to the open peer review policy of the journal. I understand that my name will be included on my report to the authors and, if the manuscript is accepted for publication, my named report including any attachments I upload will be posted on the website along with the authors' responses. I agree for my report to be made available under an Open Access Creative Commons CC-BY license (http://creativecommons.org/licenses/by/4.0/). I understand that any comments which I do not wish to

be included in my named report can be included as confidential comments to the editors, which will not be published.

I agree to the open peer review policy of the journal

To further support our reviewers, we have joined with Publons, where you can gain additional credit to further highlight your hard work (see: https://publons.com/journal/530/gigascience). On publication of this paper, your review will be automatically added to Publons, you can then choose whether or not to claim your Publons credit. I understand this statement.

Yes Choose an item.
