# Supplementary material for: PIMENTO: a primer inference toolkit to facilitate large-scale calling of amplicon sequence variants
Source: Gigascience. 2026 Jul 31;15:giag083. doi: 10.1093/gigascience/giag083 (PMC13508698; doi:10.1093/gigascience/giag083)
Supplement: Supplementary file 7 [file giag083_reviewer_1_report_revision_2.pdf]

Reviewer Report

Title: PIMENTO: A primer inference toolkit to facilitate large-scale calling of amplicon sequence variants

Version: Revision 2 **Date:** 7/6/2026

Reviewer name: Reviewer 1

Reviewer Comments to Author:

I am pleased to see that the authors have addressed the comments from the previous review.As a software package, it is important that pimento runs smoothly on commonly used platforms, particularly popular Linux distributions. I tested the software on my server running Ubuntu 24.04.4 LTS and encountered the following issues.1. Python 3.12 compatibilityUbuntu 24.04 ships with Python 3.12 by default, but pimento cannot currently be installed under Python 3.12. The installation fails with the following error:```File "<string>", line 19, in <module>ModuleNotFoundError: No module named 'pkg_resources'```Neither the manuscript nor the GitHub repository mentions this compatibility issue. At present, the only workaround is to create an environment with an older Python version (e.g., Python 3.10). I encourage the authors to improve compatibility with newer Python releases, or at least clearly document the supported Python versions and installation requirements.2. Reproducibility of the ERR14113214 exampleI attempted to reproduce the results for ERR14113214 (Table ST1) using the following command:pimento auto --strand FR -i ERR14113214.fastq -o ERR14113214 --minimum_primer_threshold 0.15However, the program reports that the option `--minimum_primer_threshold` is not recognized, and I could not find this option anywhere in the current source code.Without the option `--minimum_primer_threshold`, the results were completely different from Table ST1.In addition, I used "cutadapt" to search for the known primer sequences:cutadapt -g "GTCGGTAAAACTCGTGCCAGC...CAAACTGGGATTAGATACCCCACTATG" --revcomp -j 0 -o ERR14113214_trimmed.fastq ERR14113214.fastqThe output was:```Total reads processed: 92,642Reads with adapters: 65,282 (70.5%)Reverse-complemented: 31,921 (34.5%)```These results indicate that a substantial proportion of the reads contain the expected primer sequences.I would appreciate it if the authors could make further revisions.

Level of Interest

Please indicate how interesting you found the manuscript: Choose an item.

Quality of Written English

Please indicate the quality of language in the manuscript: Choose an item.

Declaration of Competing Interests

Please complete a declaration of competing interests, considering the following questions:

- Have you in the past five years received reimbursements, fees, funding, or salary from an organisation that may in any way gain or lose financially from the publication of this manuscript, either now or in the future?
- Do you hold any stocks or shares in an organisation that may in any way gain or lose financially from the publication of this manuscript, either now or in the future?
- Do you hold or are you currently applying for any patents relating to the content of the manuscript?
- Have you received reimbursements, fees, funding, or salary from an organization that holds or has applied for patents relating to the content of the manuscript?
- Do you have any other financial competing interests?
- Do you have any non-financial competing interests in relation to this paper?

If you can answer no to all of the above, write 'I declare that I have no competing interests' below. If your reply is yes to any, please give details below.

I declare that I have no competing interests

I agree to the open peer review policy of the journal. I understand that my name will be included on my report to the authors and, if the manuscript is accepted for publication, my named report including any attachments I upload will be posted on the website along with the authors' responses. I agree for my report to be made available under an Open Access Creative Commons CC-BY license (http://creativecommons.org/licenses/by/4.0/). I understand that any comments which I do not wish to be included in my named report can be included as confidential comments to the editors, which will not be published.

I agree to the open peer review policy of the journal

To further support our reviewers, we have joined with Publons, where you can gain additional credit to further highlight your hard work (see: https://publons.com/journal/530/gigascience). On publication of this paper, your review will be automatically added to Publons, you can then choose whether or not to claim your Publons credit. I understand this statement.

Yes Choose an item.
