# Supplementary material for: PIMENTO: a primer inference toolkit to facilitate large-scale calling of amplicon sequence variants
Source: Gigascience. 2026 Jul 31;15:giag083. doi: 10.1093/gigascience/giag083 (PMC13508698; doi:10.1093/gigascience/giag083)
Supplement: Supplementary file 8 [file giag083_reviewer_2_report_original_submission.pdf]

Reviewer Report

Title: PIMENTO: A primer inference toolkit to facilitate large-scale calling of amplicon sequence variants

Version: Original Submission **Date:** 2/1/2026

Reviewer name: Reviewer 2

Reviewer Comments to Author:

Comments:The manuscript named "PIMENTO: A PrIMEr infereNce TOolkit to facilitate large-scale calling of amplicon sequence variants" provide us a tool for the inference of the presence and identity of primers in amplicon sequencing data. I believe this is an important supplement to large-scale analysis of amplicon data from different sources for primer removal. Here are some suggestions:1.The title displays the letters PIMENTO in capital letters, which doesn't seem appropriate. It's suggested that the origin of the abbreviation be explained in the abstract or body text.2.In "2.1 Standard primer search strategy" part, there is a sentence "This library was curated from a survey of the literature, sequencing platform websites, and other resources." Details should be provided, including how many papers were investigated, the sequencing platform, and what other resources are being referred to.3.Figures 2, 3, 4, and 5 can be combined into one figure to make the content more concise, as they all use the Marine Survey Dataset for analysis and present results from different aspects. This also makes it easier for readers to understand.4.The box plot in Figure 3 can be further optimized for a more aesthetically pleasing result.5.The results in the article show that, compared with other types of samples, the differentiation effect of gut microbes is the worst when distinguishing between gut microbes, soil microbes, and marine microbes. The authors explain that this is because there are highly conserved regions at the beginning of the reads. They suggest further analysis or discussion on whether there is a way to identify these highly conserved regions to make the gut microbe samples more accurate.6.In "3.5 Primer valiation of automatically-inferred primers" part, suggest authors give an exact example for the validation of the primer inference results.

Level of Interest

Please indicate how interesting you found the manuscript: Choose an item.

Quality of Written English

Please indicate the quality of language in the manuscript: Choose an item.

Declaration of Competing Interests

Please complete a declaration of competing interests, considering the following questions:

- Have you in the past five years received reimbursements, fees, funding, or salary from an organisation that may in any way gain or lose financially from the publication of this manuscript, either now or in the future?
- Do you hold any stocks or shares in an organisation that may in any way gain or lose financially from the publication of this manuscript, either now or in the future?
- Do you hold or are you currently applying for any patents relating to the content of the manuscript?
- Have you received reimbursements, fees, funding, or salary from an organization that holds or has applied for patents relating to the content of the manuscript?
- Do you have any other financial competing interests?
- Do you have any non-financial competing interests in relation to this paper?

If you can answer no to all of the above, write 'I declare that I have no competing interests' below. If your reply is yes to any, please give details below.

I declare that i have no competing interests.

I agree to the open peer review policy of the journal. I understand that my name will be included on my report to the authors and, if the manuscript is accepted for publication, my named report including any attachments I upload will be posted on the website along with the authors' responses. I agree for my report to be made available under an Open Access Creative Commons CC-BY license (http://creativecommons.org/licenses/by/4.0/). I understand that any comments which I do not wish to be included in my named report can be included as confidential comments to the editors, which will not be published.

Choose an item.

To further support our reviewers, we have joined with Publons, where you can gain additional credit to further highlight your hard work (see: https://publons.com/journal/530/gigascience). On publication of this paper, your review will be automatically added to Publons, you can then choose whether or not to claim your Publons credit. I understand this statement.

Yes Choose an item.
