# Supplementary material for: PIMENTO: a primer inference toolkit to facilitate large-scale calling of amplicon sequence variants
Source: Gigascience. 2026 Jul 31;15:giag083. doi: 10.1093/gigascience/giag083 (PMC13508698; doi:10.1093/gigascience/giag083)
Supplement: Supplementary file 9 [file giag083_supplemental_file.docx]

Supplementary Data

**PIMENTO: A primer inference toolkit to facilitate large-scale calling of amplicon sequence variants**

# Benchmarking of PIMENTO

To benchmark the scalability of PIMENTO, a subset of 11 paired-end runs of varying read counts and file sizes was created. The read counts range from 87,792 (ERR10670995) to 15,896,076 (ERR5556694), and the file sizes range from 7.05 MB (ERR10670995) to 1.51 GB (SRR2657613), with the FASTQ files being gzipped.

For each main PIMENTO function (std, auto, are_there_primers), both forward and reverse orientation reads of all 11 runs were passed as input, and the runtime was recorded. The runtime was then plotted along with the read count and filesize, which can be seen in the following pages. The machine PIMENTO was run on for these tests is a MacBook Pro 2023 with an Apple M2 Pro chip and 16GB of RAM.

## S1: Standard primer search function

Command run:

```

pimento std -i /path/to/ERRXXX.fastq.gz -o ERRXXX -t 4

```

These bar plots show the runtime for running the *standard primer search* function. This function has been parallelised and therefore has the option of setting a threads parameter, which was set to 4 for this benchmarking. The runtime ranges from 3.71 seconds to 529 seconds. These results show that the *standard primer search* function scales and completes at a fast speed even for runs that have a filesize over 1GB and over 10 million reads, which are very large compared to typical amplicon sequencing runs.

The plots do not show a linear relationship between filesize/readcount and the runtime, which is expected due to two optimisations implemented in the algorithm. The first optimization is that a maximum of 300,000 reads are considered when searching for standard primers, which has proven to be a high enough number to confidently assess the presence of a primer. For the second efficiency, when PIMENTO collects the first 50 bases of every read (or the maximum of 300,000), unique substrings of 50 bases are stored in a counter item. Since it would be computationally unnecessary to search for primers in two identical substrings, which are expected in amplicon sequencing runs, PIMENTO only searches for primers in the unique substrings within this counter. For every match between a primer and a unique substring, the value within the counter for that substring is then incremented when computing the primer proportions. Because of this second efficiency, it is possible to have a run with fewer reads but more diversity having a higher runtime than a run with more reads but less diversity, which is what we see in these plots.

## S2: Primer cutoff prediction method

Command run:

```

pimento auto -i /path/to/ERRXXX.fastq.gz -st F -o ERRXXX

```

These bar plots show the runtime for running the *primer cutoff prediction* function. The runtime ranges from 7.98 seconds to 553 seconds. Similar to the *standard primer search* function, this method scales and completes at a fast speed even for runs that have a filesize over 1GB and over 10 million reads, without parallelisation. This function also uses the maximum read count threshold of 300 thousand reads, but doesn’t use the unique substring counter efficiency.

## S3: Are there primers function

Command run:

```

pimento are_there_primers -i /path/to/ERRXXX.fastq.gz -o ERRXXX

```

These bar plots show the runtime for running the are_there_primer utility function. The runtime ranges from 1.67 seconds to 1165.54 seconds. Similar to the previous two functions, this utility scales with the longest run taking just under 20 minutes, despite it not using the maximum read count efficiency. It does however use the unique read substring counter, which again explains the lack of linear relationship.

## S4: Running PIMENTO on Oxford Nanopore Technologies long reads

**Table ST1:** Comparison of sequences identified by the *standard primer search* strategy and the canonical primers extracted from literature by global pairwise sequence alignment for the set of ONT studies. Matched primers are either identical or almost identical in all four studies (20 runs) represented in this table. A fifth study, PRJNA1159001, was also assessed, but no standard primers were found in any of the runs tested.

| **Study** | **Run** | **Primer alignments** |
| --- | --- | --- |
| PRJEB81670**[1]** | ERR14113214 | **MiFish-U_F:**  **GCCGGTAAAACTCGTGCC Literature**  **\| \|\|\|\|\|\| \|\|\|\|\|\|\|\|**  **GTYGGTAAAWCTCGTGCCAGC Predicted**  **MiFish-UE_F**  **MiFish-U_R:**  **CATAGTGGGGTATCTAATCCCAGTTTG Literature**  **\|\|\|\|\|\|\|\|\|\|\|\|\|\|\|\|\|\|\| \|\|\|\|\|\|**  **ATAGTGGGGTATCTAATCCYAGTTTG Predicted**  **MiFish-UE_R** |
|  | ERR14113221 | **MiFish-U_F:**  **GCCGGTAAAACTCGTGCC Literature**  **\| \|\|\|\|\|\| \|\|\|\|\|\|\|\|**  **GTYGGTAAAWCTCGTGCCAGC Predicted**  **MiFish-UE_F**  **MiFish-U_R:**  **CATAGTGGGGTATCTAATCCCAGTTTG Literature**  **\|\|\|\|\|\|\|\|\|\|\|\|\|\|\|\|\|\|\| \|\|\|\|\|\|**  **ATAGTGGGGTATCTAATCCYAGTTTG Predicted**  **MiFish-UE_R** |
|  | ERR14113222 | **MiFish-U_F:**  **GCCGGTAAAACTCGTGCC Literature**  **\| \|\|\|\|\|\| \|\|\|\|\|\|\|\|**  **GTYGGTAAAWCTCGTGCCAGC Predicted**  **MiFish-UE_F**  **MiFish-U_R:**  **CATAGTGGGGTATCTAATCCCAGTTTG Literature**  **\|\|\|\|\|\|\|\|\|\|\|\|\|\|\|\|\|\|\| \|\|\|\|\|\|**  **ATAGTGGGGTATCTAATCCYAGTTTG Predicted**  **MiFish-UE_R** |
|  | ERR14114002 | **Fish-12S_F:**  **GGATTAGATACCCYACTATGY Literature**  **\|\|\|\|\|\|\|\|\|\|\|\|\|\|\|\|\|\|\|\|\|**  **GGATTAGATACCCYACTATGY Predicted**  **Fish-12S_F**  **Fish-16S_R:**  **GATTGCGCTGTTATCCCTRG Literature**  **\|\|\|\|\|\|\|\|\|\|\|\|\|\|\|\|\|\|\|\|**  **GATTGCGCTGTTATCCCTRG Predicted**  **Fish-16S_R** |
|  | ERR14114003 | **Fish-12S_F:**  **GGATTAGATACCCYACTATGY Literature**  **\|\|\|\|\|\|\|\|\|\|\|\|\|\|\|\|\|\|\|\|\|**  **GGATTAGATACCCYACTATGY Predicted**  **Fish-12S_F**  **Fish-16S_R:**  **GATTGCGCTGTTATCCCTRG Literature**  **\|\|\|\|\|\|\|\|\|\|\|\|\|\|\|\|\|\|\|\|**  **GATTGCGCTGTTATCCCTRG Predicted**  **Fish-16S_R** |
| PRJEB84473**[2]** | ERR15280011 | **MiFish-UE_F:**  **GTYGGTAAAWCTCGTGCCAGC Literature**  **\|\|\|\|\|\|\|\|\|\|\|\|\|\|\|\|\|\|\|\|\|**  **GTYGGTAAAWCTCGTGCCAGC Predicted**  **MiFish-UE_F**  **MiFish-UE_R:**  **ATAGTGGGGTATCTAATCCYAGTTTG Literature**  **\|\|\|\|\|\|\|\|\|\|\|\|\|\|\|\|\|\|\|\|\|\|\|\|\|\|**  **ATAGTGGGGTATCTAATCCYAGTTTG Predicted**  **MiFish-UE_R** |
|  | ERR15280013 | **MiFish-UE_F:**  **GTYGGTAAAWCTCGTGCCAGC Literature**  **\|\|\|\|\|\|\|\|\|\|\|\|\|\|\|\|\|\|\|\|\|**  **GTYGGTAAAWCTCGTGCCAGC Predicted**  **MiFish-UE_F**  **MiFish-UE_R:**  **ATAGTGGGGTATCTAATCCYAGTTTG Literature**  **\|\|\|\|\|\|\|\|\|\|\|\|\|\|\|\|\|\|\|\|\|\|\|\|\|\|**  **ATAGTGGGGTATCTAATCCYAGTTTG Predicted**  **MiFish-UE_R** |
|  | ERR15280019 | **MiFish-UE_F:**  **GTYGGTAAAWCTCGTGCCAGC Literature**  **\|\|\|\|\|\|\|\|\|\|\|\|\|\|\|\|\|\|\|\|\|**  **GTYGGTAAAWCTCGTGCCAGC Predicted**  **MiFish-UE_F**  **MiFish-UE_R:**  **ATAGTGGGGTATCTAATCCYAGTTTG Literature**  **\|\|\|\|\|\|\|\|\|\|\|\|\|\|\|\|\|\|\|\|\|\|\|\|\|\|**  **ATAGTGGGGTATCTAATCCYAGTTTG Predicted**  **MiFish-UE_R** |
|  | ERR15280039 | **MiFish-UE_F:**  **GTYGGTAAAWCTCGTGCCAGC Literature**  **\|\|\|\|\|\|\|\|\|\|\|\|\|\|\|\|\|\|\|\|\|**  **GTYGGTAAAWCTCGTGCCAGC Predicted**  **MiFish-UE_F**  **MiFish-UE_R:**  **ATAGTGGGGTATCTAATCCYAGTTTG Literature**  **\|\|\|\|\|\|\|\|\|\|\|\|\|\|\|\|\|\|\|\|\|\|\|\|\|\|**  **ATAGTGGGGTATCTAATCCYAGTTTG Predicted**  **MiFish-UE_R** |
|  | ERR15280067 | **MiFish-UE_F:**  **GTYGGTAAAWCTCGTGCCAGC Literature**  **\|\|\|\|\|\|\|\|\|\|\|\|\|\|\|\|\|\|\|\|\|**  **GTYGGTAAAWCTCGTGCCAGC Predicted**  **MiFish-UE_F**  **MiFish-UE_R:**  **ATAGTGGGGTATCTAATCCYAGTTTG Literature**  **\|\|\|\|\|\|\|\|\|\|\|\|\|\|\|\|\|\|\|\|\|\|\|\|\|\|**  **ATAGTGGGGTATCTAATCCYAGTTTG Predicted**  **MiFish-UE_R** |
| PRJNA1418494**[3]** | SRR37117551 | **MiFish-U_F:**  **GCCGGTAAAACTCGTGCC Literature**  **\| \|\|\|\|\|\| \|\|\|\|\|\|\|\|**  **GTYGGTAAAWCTCGTGCCAGC Predicted**  **MiFish-UE_F**  **MiFish-U_R:**  **CATAGTGGGGTATCTAATCCCAGTTTG Literature**  **\|\|\|\|\|\|\|\|\|\|\|\|\|\|\|\|\|\|\| \|\|\|\|\|\|**  **ATAGTGGGGTATCTAATCCYAGTTTG Predicted**  **MiFish-UE_R** |
|  | SRR37117554 | **MiFish-U_F:**  **GCCGGTAAAACTCGTGCC Literature**  **\| \|\|\|\|\|\| \|\|\|\|\|\|\|\|**  **GTYGGTAAAWCTCGTGCCAGC Predicted**  **MiFish-UE_F**  **MiFish-U_R:**  **CATAGTGGGGTATCTAATCCCAGTTTG Literature**  **\|\|\|\|\|\|\|\|\|\|\|\|\|\|\|\|\|\|\| \|\|\|\|\|\|**  **ATAGTGGGGTATCTAATCCYAGTTTG Predicted**  **MiFish-UE_R** |
|  | SRR37117558 | **MiFish-U_F:**  **GCCGGTAAAACTCGTGCC Literature**  **\| \|\|\|\|\|\| \|\|\|\|\|\|\|\|**  **GTYGGTAAAWCTCGTGCCAGC Predicted**  **MiFish-UE_F**  **MiFish-U_R:**  **CATAGTGGGGTATCTAATCCCAGTTTG Literature**  **\|\|\|\|\|\|\|\|\|\|\|\|\|\|\|\|\|\|\| \|\|\|\|\|\|**  **ATAGTGGGGTATCTAATCCYAGTTTG Predicted**  **MiFish-UE_R** |
|  | SRR37117559 | **MiFish-U_F:**  **GCCGGTAAAACTCGTGCC Literature**  **\| \|\|\|\|\|\| \|\|\|\|\|\|\|\|**  **GTYGGTAAAWCTCGTGCCAGC Predicted**  **MiFish-UE_F**  **MiFish-U_R:**  **CATAGTGGGGTATCTAATCCCAGTTTG Literature**  **\|\|\|\|\|\|\|\|\|\|\|\|\|\|\|\|\|\|\| \|\|\|\|\|\|**  **ATAGTGGGGTATCTAATCCYAGTTTG Predicted**  **MiFish-UE_R** |
|  | SRR37117560 | **MiFish-U_F:**  **GCCGGTAAAACTCGTGCC Literature**  **\| \|\|\|\|\|\| \|\|\|\|\|\|\|\|**  **GTYGGTAAAWCTCGTGCCAGC Predicted**  **MiFish-UE_F**  **MiFish-U_R:**  **CATAGTGGGGTATCTAATCCCAGTTTG Literature**  **\|\|\|\|\|\|\|\|\|\|\|\|\|\|\|\|\|\|\| \|\|\|\|\|\|**  **ATAGTGGGGTATCTAATCCYAGTTTG Predicted**  **MiFish-UE_R** |
| PRJNA1389880**[4]** | SRR36479744 | **MiFish-U_F:**  **GCCGGTAAAACTCGTGCC Literature**  **\|\|\|\|\|\|\|\|\|\|\|\|\|\|\|\|\|\|**  **GCCGGTAAAACTCGTGCC Predicted**  **MiFish-U_F**  **MiFish-U_R:**  **CATAGTGGGGTATCTAATCCCAGTTTG Literature**  **\|\|\|\|\|\|\|\|\|\|\|\|\|\|\|\|\|\|\| \|\|\|\|\|\|**  **ATAGTGGGGTATCTAATCCYAGTTTG Predicted**  **MiFish-UE_R** |
|  | SRR36479749 | **MiFish-U_F:**  **GCCGGTAAAACTCGTGCC Literature**  **\|\|\|\|\|\|\|\|\|\|\|\|\|\|\|\|\|\|**  **GCCGGTAAAACTCGTGCC Predicted**  **MiFish-U_F**  **MiFish-U_R:**  **CATAGTGGGGTATCTAATCCCAGTTTG Literature**  **\|\|\|\|\|\|\|\|\|\|\|\|\|\|\|\|\|\|\| \|\|\|\|\|\|**  **ATAGTGGGGTATCTAATCCYAGTTTG Predicted**  **MiFish-UE_R** |
|  | SRR36479750 | **MiFish-U_F:**  **GCCGGTAAAACTCGTGCC Literature**  **\|\|\|\|\|\|\|\|\|\|\|\|\|\|\|\|\|\|**  **GCCGGTAAAACTCGTGCC Predicted**  **MiFish-U_F**  **MiFish-U_R:**  **CATAGTGGGGTATCTAATCCCAGTTTG Literature**  **\|\|\|\|\|\|\|\|\|\|\|\|\|\|\|\|\|\|\| \|\|\|\|\|\|**  **ATAGTGGGGTATCTAATCCYAGTTTG Predicted**  **MiFish-UE_R** |
|  | SRR36479753 | **MiFish-U_F:**  **GCCGGTAAAACTCGTGCC Literature**  **\|\|\|\|\|\|\|\|\|\|\|\|\|\|\|\|\|\|**  **GCCGGTAAAACTCGTGCC Predicted**  **MiFish-U_F**  **MiFish-U_R:**  **CATAGTGGGGTATCTAATCCCAGTTTG Literature**  **\|\|\|\|\|\|\|\|\|\|\|\|\|\|\|\|\|\|\| \|\|\|\|\|\|**  **ATAGTGGGGTATCTAATCCYAGTTTG Predicted**  **MiFish-UE_R** |
|  | SRR36479756 | **MiFish-U_F:**  **GCCGGTAAAACTCGTGCC Literature**  **\|\|\|\|\|\|\|\|\|\|\|\|\|\|\|\|\|\|**  **GCCGGTAAAACTCGTGCC Predicted**  **MiFish-U_F**  **MiFish-U_R:**  **CATAGTGGGGTATCTAATCCCAGTTTG Literature**  **\|\|\|\|\|\|\|\|\|\|\|\|\|\|\|\|\|\|\| \|\|\|\|\|\|**  **ATAGTGGGGTATCTAATCCYAGTTTG Predicted**  **MiFish-UE_R** |

[1]	K. Doorenspleet et al., “The Long and the Short of It: Nanopore‐Based	eDNA	Metabarcoding of Marine Vertebrates Works; Sensitivity and Species‐Level Assignment Depend on Amplicon Lengths,” Molecular Ecology Resources, vol. 25, no. 4, Feb. 2025, doi: 10.1111/1755-0998.14079.

[2]	L. Jansen et al., “A Baseline of Fish Species Richness Through	eDNA	Metabarcoding in an Understudied Tropical Mangrove Coast of Java, Indonesia,” Environmental DNA, vol. 7, no. 6, Nov. 2025, doi: 10.1002/edn3.70201.

[3]	L. Cartairade, J. Poulain, Q. Carradec, S. Mangenot, P. Wincker, and S. Planes, “Optimizing a Novel	eDNA	‐Based Framework for Reef Fish Biodiversity Monitoring Using an Autonomous Filtration System and in situ Nanopore Sequencing,” Ecology and Evolution, vol. 16, no. 3, Mar. 2026, doi: 10.1002/ece3.73254.

[4]	N. K. D. Cahyani et al., “eDNA	Metabarcoding as a Promising Conservation Tool to Monitor Fish Diversity in Indonesia Marine Protected Areas,” Environmental

NA, vol. 8, no. 1, Jan. 2026, doi: 10.1002/edn3.702
